# Leukocyte subtyping predicts for treatment failure and poor survival in anal squamous cell carcinoma

**DOI:** 10.1186/s12885-022-09742-7

**Published:** 2022-06-24

**Authors:** Daniel R. Principe, Jose L. Cataneo, Kaytlin E. Timbers, Regina M. Koch, Klara Valyi-Nagy, Anders Mellgren, Ajay Rana, Gerald Gantt

**Affiliations:** 1 Medical Scientist Training Program, University of Illinois College of Medicine, Chicago, IL USA; 2 Department of Surgery, University of Illinois at Chicago, IL Chicago, USA; 3 Department of Pathology, University of Illinois at Chicago, IL Chicago, USA; 4 Department of Surgery, Division of Colorectal Surgery, University of Illinois at Chicago, Chicago, IL, USA; 5 Jesse Brown VA Medical Center, Chicago, IL USA

**Keywords:** Anal Cancer, Biomarkers, Chemotherapy, Radiation, Immunology, Tumor Microenvironment

## Abstract

**Background:**

Anal squamous cell carcinoma (SCC) generally carries a favorable prognosis, as most tumors are highly sensitive to standard of care chemoradiation. However, outcomes are poor for the 20–30% of patients who are refractory to this approach, and many will require additional invasive procedures with no guarantee of disease resolution.

**Methods:**

To identify the patients who are unlikely to respond to the current standard of care chemoradiation protocol, we explored a variety of objective clinical findings as a potential predictor of treatment failure and/or mortality in a single center retrospective study of 42 patients with anal SCC.

**Results:**

Patients with an increase in total peripheral white blood cells (WBC) and/or neutrophils (ANC) had comparatively poor clinical outcomes, with increased rates of death and treatment failure, respectively. Using pre-treatment biopsies from 27 patients, tumors with an inflamed, neutrophil dominant stroma also had poor therapeutic responses, as well as reduced overall and disease-specific survival. Following chemoradiation, we observed uniform reductions in nearly all peripheral blood leukocyte subtypes, and no association between peripheral white blood cells and/or neutrophils and clinical outcomes. Additionally, post-treatment biopsies were available from 13 patients. In post-treatment specimens, patients with an inflamed tumor stroma now demonstrated improved overall and disease-specific survival, particularly those with robust T-cell infiltration.

**Conclusions:**

Combined, these results suggest that routinely performed leukocyte subtyping may have utility in risk stratifying patients for treatment failure in anal SCC. Specifically, pre-treatment patients with a high WBC, ANC, and/or a neutrophil-dense tumor stroma may be less likely to achieve complete response using the standard of care chemoradiation regimen, and may benefit from the addition of a subsequent line of therapy.

**Supplementary Information:**

The online version contains supplementary material available at 10.1186/s12885-022-09742-7.

## Introduction

Anal squamous cell carcinomas (SCC) are HPV-associated cancers most frequently affecting the immunocompromised [1]. Though rare, the incidence of anal SCC has been steadily increasing in recent years, particularly among select populations including women, persons living with HIV, and transplant patients [2]. Locally advanced anal SCC carries a favorable prognosis largely due to the advent of the Nigro protocol, a multimodal therapy regimen combining external beam radiation, 5-fluorouracil (5-FU), and mitomycin-C [3]. This approach has been the standard of care for anal SCC for decades, with a majority of patients achieving complete responses [4]. However, for the 20–30% of patients who fail to achieve complete responses with first-line therapy, prognosis is particularly poor [5], and without additional intervention, patients with persistent disease carry a 2-year survival rate of 28% and a 5-year survival rate of 0% [6].

For persistent or recurrent disease, abdominoperineal resection (APR) is generally indicated as salvage treatment, with inguinal lymph node dissection if indicated. However, APR is a highly invasive procedure, associated with a high rate of wound complications, as well as sexual and urinary dysfunction [6, 7]. While this can achieve disease control for some patients, others will continue to experience disease progression and prognosis is variable [6, 8]. For patients with inoperable disease, there is currently no consensus treatment approach. Most will be treated with subsequent-line chemotherapy, though median survival remains a dismal 12 months [1, 9]. Hence, there is an urgent need to identify the patients unlikely to achieve satisfactory disease control using the current standard of care in hopes of providing a more effective first-line therapy, thereby limiting the need for invasive or poorly effective treatments and improving long-term survivability.

Though HPV-status has been shown to predict clinical outcomes, HPV infections are ubiquitous in anal SCC, affecting as many as 90% of patients [10–13]. Hence, while HPV-positive patients have an improved prognosis, HPV status has limited utility as a prognostic biomarker. Recent evidence suggests that alterations in peripheral blood biomarkers may have utility in predicting disease-related mortality [14]. Specifically, lower hemoglobin and elevated peripheral white blood cells have been associated with poor survival [14]. Other studies have arrived at similar conclusions, suggesting that patients with pre-treatment leukocytosis and/or neutrophilia may also have poor outcomes [15–18]. We therefore evaluated a cohort of 42 patients with previously untreated, non-metastatic anal SCC and related alterations in peripheral as well as tumor-infiltrating leukocyte subtypes to primary therapy success and overall survival. Using this approach, we found that patients with a relative increase in total peripheral white blood cells and/or absolute neutrophil count had comparatively poor clinical outcomes, with increased rates of death and treatment failure, respectively.

Additionally, using pre-treatment tissue biopsies, we determined that patients with an inflamed tumor stroma also had poor clinical outcomes irrespective of HPV status, particularly patients with an increase in tumor-infiltrating neutrophils. After completing first-line chemoradiation, we observed a ubiquitous decrease in all peripheral leukocyte subtypes, though none had a significant relationship with therapy response or survival. In post-treatment biopsies, patients with an inflamed tumor stroma had improved clinical outcomes, particularly those with a high degree of T-cell infiltration. Hence, the assessment of pre- and post-treatment peripheral and/or tumor-infiltrating leukocyte subtypes warrants continued investigation as a prognostic biomarker in patients undergoing first-line chemoradiation for anal SCC.

## Materials & methods

### Patients, follow-up, and sample collection

Banked anal SCC tissues were provided by the University of Illinois Health Biorepository. All tissues were collected from patients over 18 years of age with anal SCC, who were invited to participate when seen at the University of Illinois Hospital (UIC), and for each, fully informed, written consent was obtained in accordance with local IRB guidelines. Patients were initially evaluated during an outpatient consult, including a clinical examination of their tumor and collection of basic demographic information. At this point, biopsies were obtained and sent to pathology for definitive diagnosis. Remaining tissues were banked as described, and used for all subsequent analyses in this study in a retrospective manner. Patients received the current standard of care chemoradiation regimen consisting of 5-FU administered on days 1–4 and 29–32, mitomycin-C on days 1 and 29, and a total dose of 45 to 50.4 Gy radiation delivered in approximately 25 sessions over 5–6 weeks. Adjustments were made as needed on a case-by-case basis in accordance with the National Comprehensive Cancer Network (NCCN) guidelines. Blood was collected prior to the first dose of chemotherapy or radiation, and complete blood counts (CBC) with differential conducted by the University of Illinois Hospital clinical laboratory staff using Beckman Coulter hematology analyzers. On the last day of treatment, blood was again collected for the post-treatment CBC with differential. Following the last day of treatment, for patients with residual disease, post-treatment biopsies were obtained if clinically indicated and analyzed as described.

To determine the primary therapy outcome, if patients achieved clinical remission during or after completing treatment, and had no evidence of recurrence during the follow-up period, they were classified as having responded to chemoradiation. However, if patients either had (1) persistent disease following therapy that did not regress or (2) clinical recurrence at any point during the follow up period after initially showing no evidence of disease, they were classified as non-responders.

### Exclusion criteria

Patients were excluded if they had received a prior line of therapy, were previously enrolled in a different study, or lost to follow-up.

### Histology and immunohistochemistry

Tissues were fixed in 10% formalin, paraffin-embedded, and sections at 4 mm interval were cut from each tissue, and stained with hematoxylin and eosin (H&E) or via immunohistochemistry (IHC). For immunohistochemistry, slides were deparaffinized by xylenes and rehydrated by ethanol gradient, then heated in a pressure cooker using DAKO retrieval buffer (DAKO, Santa Clara, CA). Endogenous peroxidases were quenched in 3% hydrogen peroxide in methanol for 30 min. Tissues were blocked with 0.5% BSA in PBS for 30 min and incubated with primary antibodies against: P16, CD45, CD3, CD68, or Neutrophil Elastase (abcam, Cambridge, MA) 1:100–1:200 overnight at 4 °C. Slides were developed using HRP-conjugated secondary antibodies followed by DAB substrate/buffer (DAKO, Santa Clara, CA).

### Tissue slide counts, scores, and measurements

All counts were performed as described previously [19, 20]. In brief, tissues were quantified by a minimum of three blinded investigators and each value displayed includes the average of a minimum of three high power fields per specimen. All counts from each investigator were averaged and value distributions were visualized via Minitab express software, showing the median value as a solid line as well as all individual values.

### Microscopy

All images were acquired using a Nikon 40x-400 × Epi-Fluorescent Inverted Microscope with Phase Contrast Kit and Nikon brightfield camera attachment. Negative slides were used for white balance, and for all images no analog or digital gain was used. LUTs were used to reduce background based on negative control slides. These LUT values and exposure times were standardized and used for all other similarly stained slides.

### Authentication of key resources

All antibodies are commercially available and validated by the manufacturer for the specific applications used in this study.

### Statistical analysis

Data were analyzed by either student’s T test or ANOVA fit to a general linear model in Minitab express, the validity of which was tested by adherence to the normality assumption and the fitted plot of the residuals. Results were arranged by the Tukey method. Time-to-event survival data was evaluated via the Kaplan–Meier method [21, 22]. Data were considered significant at *p* < 0.05 unless otherwise noted.

### Study approval

This study was conducted following local IRB approval from the University of Illinois at Chicago. Patient specimens were obtained from fully consenting patients in a de-identified manner from the tissue bio-repository at the University of Illinois at Chicago following local IRB approval.

### Patient and public involvement statement

Beyond offering informed, written consent to collect tissues for research purposes as described above, neither our patients nor the public were involved in this study.

### Guide statement

All methods were carried out in accordance with relevant guidelines and regulations.

## Results

### Increased peripheral blood leukocytes or circulating neutrophils predict poor outcomes in anal SCC patients

To identify potential predictors of treatment failure and/or mortality, we evaluated a total of 42 patients with non-metastatic anal SCC (Tables S1 and S2). Of these patients, 61.9% were male and 38.1% female (Figure S1A). Additionally, 57.1% identified as black/African American, 31% identified as white/Caucasian, and the remaining 11.9% as a different racial group, either Hispanic or Asian American (Figure S1A). Half of patients had a history of HIV infection (Figure S1A), and 81% were able to achieve complete responses with the standard of care chemoradiation protocol, with the remaining 19% showing persistent disease (Figure S1B). At the end of the follow-up period, we observed an overall survival of 66.7% (Figure S1C), and following the exclusion of 5 patients who died of causes other than anal SCC, a disease specific survival rate of 75.7% (Figure S1D). Additionally, 76.4% of patients with complete responses were alive at the study endpoint compared to only 25% of patients who demonstrated persistent disease (Figure S1E). This corresponded to a disease-specific survival rate of 86.7% in patients showing complete responses to first-line chemoradiation, and a disease-specific survival rate of 28.6% for patients with persistent disease (Figure S1F).

HIV status had no bearing on primary therapy outcome (Figure S1G), though black/African American patients had poorer response rates than all other groups, with 29.2% failing to achieve complete responses compared to 0% in white/Caucasian patients and 0% in 5 patients belonging to other racial groups (Figure S1H). Patients positive for HIV had a comparative increase in all cause mortality (Figure S1I), as did black/African American patients, who had an overall survival rate of 54.2% compared to 84.6% for white/Caucasian patients and 80% for patients belonging to other racial groups (Figure S1J). We observed similar results with disease-specific survival, with slightly worse outcomes for patients with HIV (Figure S1K), as well as for black/African American patients (Figure S1L). Natal sex had no bearing on clinical outcomes (Figure S2), nor did tumor stage (Figure S3). Additional factors including age and smoking status were also not significantly associated with outcome.

**Fig. 1:**
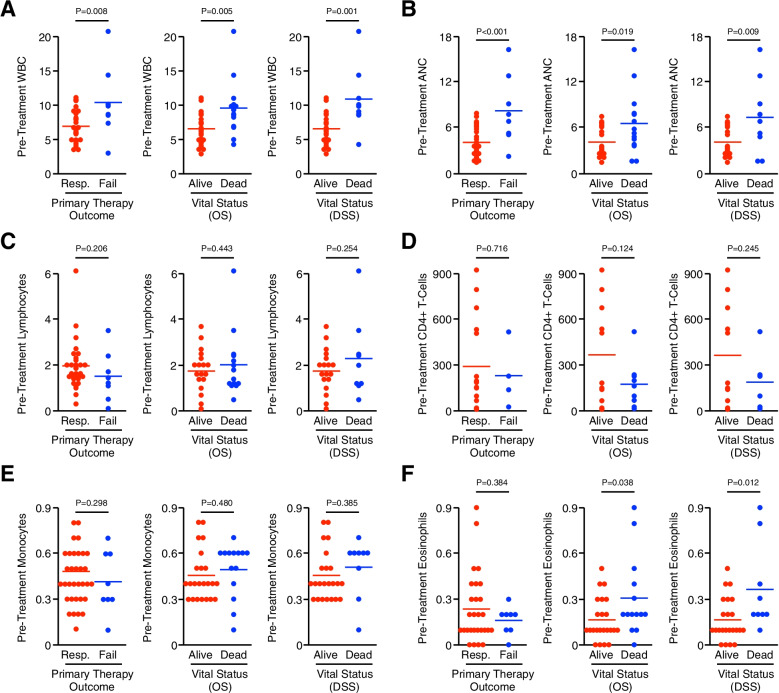
Increased peripheral blood leukocytes or circulating neutrophils predict poor outcomes in anal SCC patients. Peripheral blood specimens were collected from 42 chemo-naïve patients with anal SCC and subjected to routine complete blood count (CBC) with differential. Using these data, the number of (**A**) total white blood cells (WBCs), (**B**) absolute neutrophil count (ANC), (**C**) lymphocytes, (**D**) CD4 + T-cells for the 21 HIV + patients, (**E**) monocytes, and (**F**) eosinophils were related to clinical outcomes in the form of primary therapy outcome, overall survival (OS), and disease-specific survival (DSS). Data are presented as an individual value plot and considered significant at *p* < 0.05

To explore the potential prognostic utility of routinely performed peripheral blood leukocyte subtypes, we next evaluated pre-treatment complete blood counts (CBC) with differential taken before treatment was initiated and related these data to both primary therapy outcome and overall survival status. We found that patients who either failed to respond to therapy or died within the follow-up period had a significant increase in the number of total circulating white blood cells (WBCs), though there was no significant difference in WBCs between patients of differing sex, race, HIV status, or disease stage (Fig. 1A and S4A). We observed similar results using absolute neutrophil counts (ANC), with a more significant increase in the ANC of patients who failed to respond to first-line chemoradiation, and a more modest though still significant increase in the ANC of patients who died during follow-up (Fig. 1B). Again, there was no significant difference between the ANC of patients of differing sex, race, HIV status, or disease stage (Fig. 1B and S4B). We also evaluated alterations in peripheral lymphocytes, CD4 + T-cells for patients with HIV, as well as circulating monocytes, eosinophils, and basophils, none of which were predictive for therapy response, overall, or disease-specific survival (Fig. 1C-F and S4C).

### Patients with a peripheral WBC count of ≥ 8 or an ANC ≥ 5 have poor therapy response rates and reduced survival

**Fig. 2:**
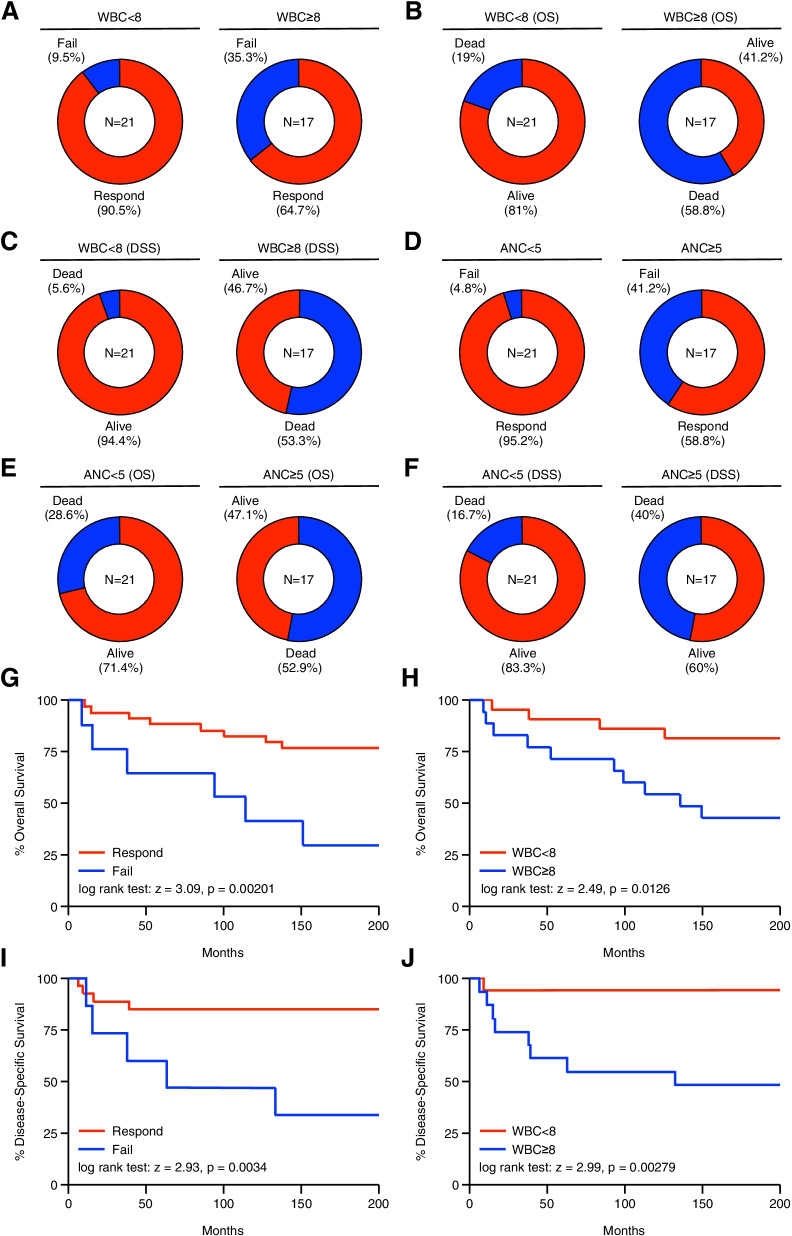
Patients with a peripheral WBC count of ≤ 8 or an ANC ≤ 5 have poor therapy response rates and reduced survival. Peripheral blood specimens were collected from 42 chemo-naïve patients with anal SCC, and subjected to routine complete blood count (CBC) with differential. Patients were separated into two groups: those with a total peripheral white blood cell (WBC) count of < 8, and those with a WBC count of ≥ 8. These groups were compared for (**A**) primary therapy outcome, (**B**) overall survival (OS), and (**C**) disease-specific survival. Also using results of the CBC with differential, patients were evaluated for absolute neutrophil count (ANC), and arranged as having an ANC of < 5 or an ANC of ≥ 5. These groups were similarly evaluated for (**D**) primary therapy outcome, (**E**) overall survival (OS), and (**F**) disease-specific survival. (**G,H**) Kaplan–Meier curve indicating months of overall survival for patients arranged by primary therapy response or WBC count of above or below 8. (**I,J**) Kaplan–Meier curve indicating months of disease-specific survival for patients arranged by primary therapy response or a WBC count of above or below 8

To relate the above findings to a clinically useful means of risk stratifying patients for poor clinical outcomes, we created cut off values for WBC (≥ 8) and ANC (≥ 5) using the median values rounded up to the nearest whole number, and evaluated clinical outcomes for patients above or below these thresholds (both shown as × 1,000/uL). Using this approach, we determined that patients with a WBC of < 8 had a treatment failure rate of 9.5% compared to 35.3% for patients with a WBC count of ≥ 8 (Fig. 2A). WBC was a highly accurate predictor of overall survival, with patients having a WBC count of < 8 having an overall survival rate of 81% compared to 41.2% for patients with a WBC count of ≥ 8 (Fig. 2B). This was also observed using disease specific survival, as patients having a WBC count of < 8 having 94.4% disease specific survival compared to 46.7% for patients with a WBC count of ≥ 8 (Fig. 2C). We conducted a similar analysis using pre-treatment ANC values, and a cut off value of ≥ 5. We found that patients with an ANC of < 5 had a treatment failure rate of 4.8% compared to 41.2% for patients with an ANC of ≥ 5 (Fig. 2D). This corresponded to a relative increase in mortality, with an overall survival rate of 71.4% for patients with an ANC of < 5, and 47.1% for patients with an ANC of ≥ 5 (Fig. 2E), and disease specific survival rates of 83.3% and 60%, respectively (Fig. 2F).

We next evaluated the utility of this approach at predicting overall survival (starting at the time treatment was initiated) using the Kaplan–Meier method. While there was no statistically significant relationship between sex, race, or HIV status and survival (Figure S5A-F), patients who demonstrated complete responses had far better overall survival than those who experienced persistent disease (Fig. 2G). Stratifying patients by total peripheral WBCs produced a similar curve, as patients having a WBC count of < 8 showed a highly significant survival advantage compared to those with a WBC count of ≥ 8 (Fig. 2H), though this was not observed using an ANC cut off of value of ≥ 5 (Figure S5G). We observed similar results regarding disease-specific survival, which was closely related to having a therapy response and WBC count, though there was no statistically significant relationship between disease-specific survival and ANC (Fig. 2I,J and S5H).

### Pre-treatment anal SCC tumors with an inflamed, neutrophil-dominant stroma are associated with poor therapeutic responses and reduced overall survival

**Fig. 3:**
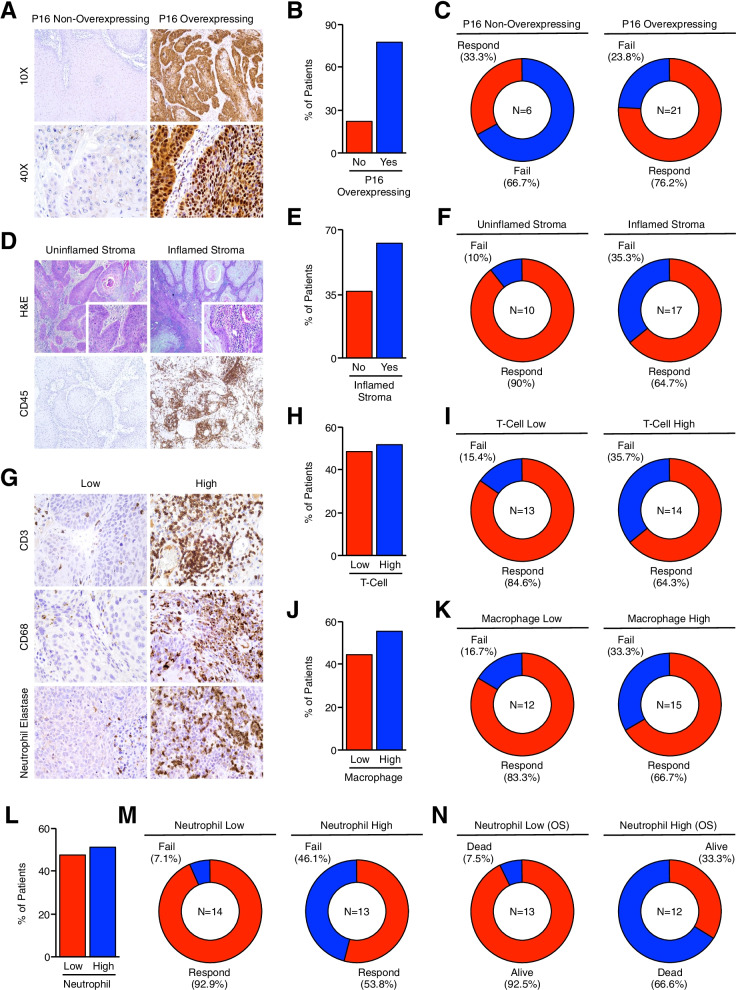
Pre-treatment anal SCC tumors with an inflamed, neutrophil-dominant stroma are associated with poor therapeutic responses and reduced overall survival. (**A**) Excisional biopsies from 27 chemo-naïve anal SCC patients were sectioned and stained via immunohistochemistry for the HPV surrogate marker p16INK4a (P16), and representative images shown for P16-overexpressing and P16 non-overexpressing tumors. (**B**) The percent of tissue specimens with and without P16 overexpression. (**C**) Patients were separated as being P16-overexpressing (P16 +) or P16 non-overexpressing (P16-) and primary therapy outcome shown as a pie chart. (**D-F**) Tissues were stained with H&E or via immunohistochemistry for the pan-leukocyte antigen CD45. Tumors comprised of ≤ 40% leukocytes categorized as having an inflamed stroma, which was then related to primary therapy outcome. (**G**) Tumor sections were stained for CD3-positive T-cells, CD68-positive macrophages, and neutrophils (neutrophil elastase-positive cells). Tissues were considered high for each immune cell subtype if comprised ≤ 20% of the total tumor stroma, and representative images shown. (**H,I**) The percent of patients with T-cell high and T-cell low tumors, as well as therapy responses for each group. (**J,K**) The percent of patients with macrophage high and macrophage low tumors, as well as therapy responses for each group. (**L**) The percent of patients with neutrophil high and neutrophil low tumors, which was then related to (**M**) primary therapy outcome and (**N**) overall survival

Of our 42 patients, pre-treatment biopsies were available from 27. Using these tissue specimens, we next evaluated alterations in tumor-infiltrating leukocyte subtypes and related them to clinical outcomes. As HPV status has been shown to predict for therapy response, we first determined the HPV status of our patients using p16INK4a (P16) overexpression as a surrogate marker of HPV infection (Fig. 3A). Using this approach, 21/27 (77.8%) patients demonstrated P16 overexpression (Fig. 3B), and P16-overexpressing patients had improved therapy response rates as well as a modest improvement in disease specific survival (Fig. 3C and S6A). We subsequently evaluated the degree of leukocyte infiltration within the tumor stroma by H&E staining, which was confirmed by immunohistochemical staining for the pan-leukocyte antigen CD45. Though nearly all patients had some degree of leukocyte infiltration, 17/27 patients (63%) had a stroma comprised of ≥ 40% leukocytes, and were categorized as having an inflamed tumor stroma (Fig. 3D,E). These patients had a higher rate of treatment failure compared to those with an uninflamed stroma (Fig. 3F), with worse overall and disease specific survival (Figure S6B).

We next examined alterations in tumor-infiltrating leukocyte subtypes, specifically CD3-positive T-cells, CD68-positive macrophages, and neutrophils (neutrophil elastase-positive cells) (Fig. 3G). Fourteen of 27 (51.9%) tumor specimens had a CD3 + T-cell infiltrate composing ≥ 20% of the tumor stroma and were categorized as being T-cell high, and the remaining 13/27 (48.1%) were categorized as T-cell low (Fig. 3H). Though T-cell high patients had a modest increase in treatment failure compared to T-cell low patients (Fig. 3I), there was no statistically significant difference in overall or disease-specific survival rates (Figure S6C). The 15/27 (55.6%) patients with macrophage high tumors had a modest increase in the rate of treatment failure (Fig. 3J,K), as well as worse overall survival and disease specific survival rates (Figure S6D). Thirteen of 27 patients (48.1%) were neutrophil high, and the remaining 14/27 (51.9%) neutrophil low (Fig. 3L). Patients with neutrophil high tumors had a highly significant decrease in primary treatment responses, with a 46.1% failure rate compared to 7.1% for neutrophil low tumors (Fig. 3M). This corresponded to a significant decrease in overall survival rate (33.3% for neutrophil high tumors compared to 92.5% for neutrophil low tumors), with similar results using disease specific survival (Fig. 3N and S6E).

### Increased tumor-infiltrating neutrophils predict for poor overall and disease specific survival in chemo-naïve anal SCC patients

**Fig. 4:**
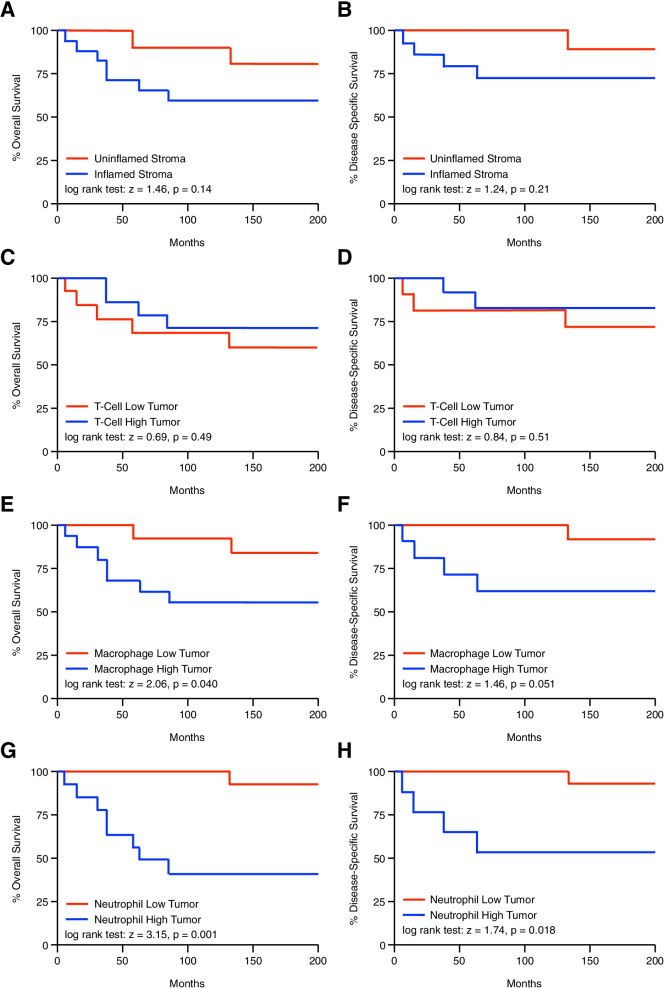
Increased tumor-infiltrating neutrophils predict for poor overall and disease specific survival in chemo-naïve anal SCC patients. Kaplan–Meier curve indicating months of overall or disease-specific survival for pre-treatment anal SCC patients arranged by (**A,B**) an inflamed or uninflamed tumor stroma, or the degree of tumor-infiltrating (**C,D**) T-cells, (**E,F**) macrophages, or (**G,H**) neutrophils

Using these data, we next evaluated the relationship between the pre-treatment immune microenvironment and months of patient survival by the Kaplan–Meier method. We found no statistically significant difference in months of overall or disease specific survival for patients with an inflamed versus uninflamed stroma (Fig. 4A,B), with similar results for patients with T-cell low or T-cell high tumors (Fig. 4C,D). While patients with high tumor-infiltrating macrophages had worse overall survival, this was near the threshold for statistical significance and the relationship to disease specific survival was not significant (Fig. 4E,F). However, patients with high tissue-infiltrating neutrophils had significantly worse overall and disease specific survival compared to those with limited neutrophil involvement (Fig. 4G,H). Patients with high neutrophil infiltration demonstrated a median overall survival of 60.5 months and a median disease specific survival of 132.5 months compared to 200 months of overall and disease specific survival for patients with low neutrophil infiltration (Fig. 4G,H).

### Following chemoradiation, anal SCC tumors with an inflamed, T-cell-dominant stroma are associated with improved therapeutic responses and better overall survival

Given the well-documented effects of chemotherapy on immune function [23], we subsequently explored post-treatment leukocyte counts and their relationship to clinical outcomes. Following completion of standard of care chemoradiation, we observed a highly significant decrease in peripheral WBCs, also reflected by post-treatment ANC, lymphocytes, and CD4 + T-cells for HIV patients (Figure S7A). We then related these post-treatment values to clinical outcomes, and unlike pre-treatment values, found that neither post-treatment WBCs nor ANC had any relationship to primary therapy outcome, with similar results observed for all other leukocyte subtypes (Figure S7B). We observed similar results with respect to vital status, with no observable relationship between any leukocyte quantification and overall or disease-specific survival (Figure S7C,D).

**Fig. 5:**
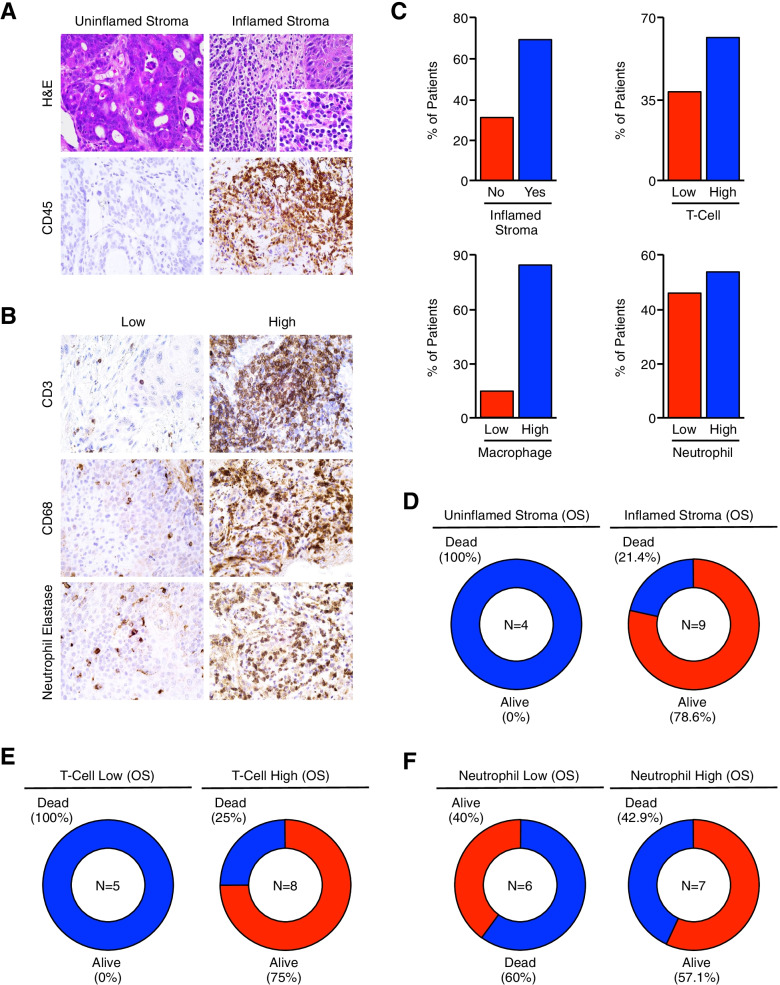
Following chemoradiation, anal SCC tumors with an inflamed, T-cell-dominant stroma are associated with improved therapeutic responses and better overall survival. (**A**) Following completion of chemoradiation, biopsies from 13 anal SCC patients were sectioned and stained either with H&E or via immunohistochemistry for the pan-leukocyte antigen CD45. As previously, tumors comprised of ≤ 40% leukocytes categorized as having an inflamed stroma. (**B**) Tissues were also stained for CD3-positive T-cells, CD68-positive macrophages, and neutrophils (neutrophil elastase-positive cells). Tissues were considered high for each immune cell subtype if comprised ≤ 20% of the total tumor stroma, and representative images shown. (**C**) The percent of patients with an inflamed or uninflamed stroma, a T-cell low or T-cell high tumor, a macrophage low or macrophage high tumor, or a neutrophil low or neutrophil high tumor. (**D**) Overall survival for patients with an inflamed or uninflamed post-treatment tumor stroma. (**E,F**) Overall survival arranged by the degree of post-treatment T-cell or neutrophil infiltration

Of the initial 42 patients, post-treatment biopsies were available from 13. We next assessed the composition of the immune microenvironment as previously described, staining tumor specimens either with H&E, or by immunohistochemistry for the pan-leukocyte antigen CD45, T-cell marker CD3, macrophage marker CD68, or neutrophil elastase (Fig. 5A,B). By the previous criteria, 9/13 (69.2%) patients had an inflamed tumor stroma, 8/13 (61.5%) were classified as T-cell high, 11/13 (84.6%) as macrophage high, and 7/13 (53.8%) neutrophil high (Fig. 5C). Contrasting our results using pre-treatment specimens, patients with an inflamed tumor stroma had improved clinical outcomes, with an overall survival rate of 78.6% compared to 0% for the patients with an uninflamed stroma (Fig. 5D). We observed similar results for patients with T-cell high tumors, where overall survival was 75% compared to 0% for T-cell low patients (Fig. 5E). As nearly all patients were macrophage high, there was no significant relationship between macrophage infiltration and survival. However, also contradicting our pre-treatment observations, tumor-infiltrating neutrophils had no significant relationship to overall survival, with an overall survival rate of 40% for neutrophil low tumors and 57.1% for neutrophil high tumors (Fig. 5F).

### Increased post-treatment tumor-infiltrating lymphocytes are associated with improved overall and disease-specific survival in anal SCC

**Fig. 6:**
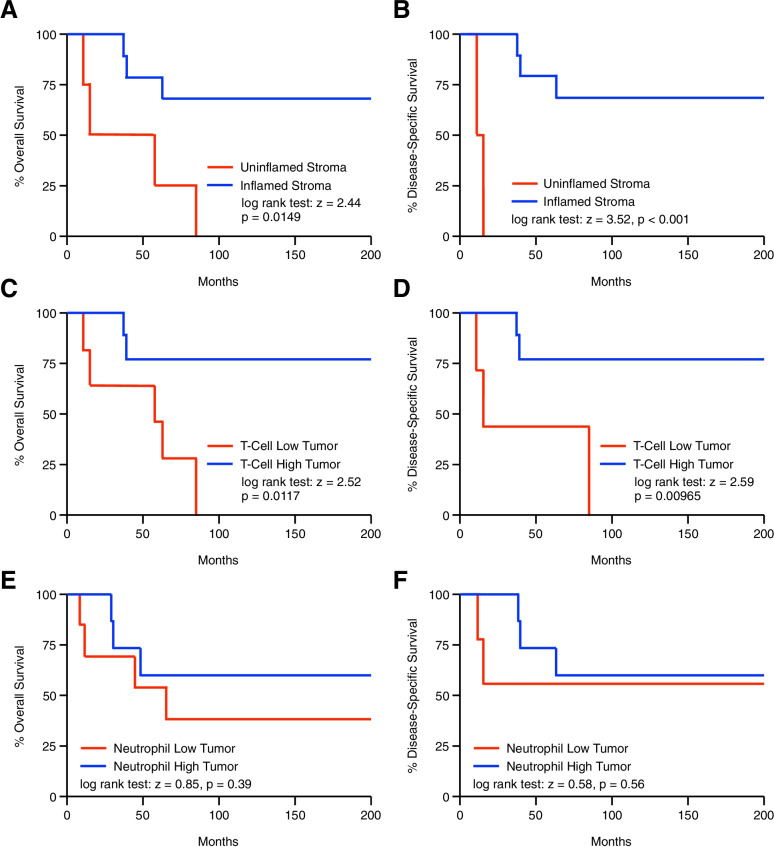
Increased post-treatment tumor-infiltrating lymphocytes are associated with improved overall and disease-specific survival in anal SCC. **(A)** Kaplan–Meier curve indicating months of overall or disease-specific survival for post-treatment anal SCC patients arranged by (**A,B**) an inflamed or uninflamed tumor stroma, or the degree of tumor-infiltrating (**C,D**) T-cells or (**E,F**) neutrophils

We finally evaluated alterations in the post-treatment immune microenvironment and survival by the Kaplan–Meier method. Contrasting our observations using pre-treatment specimens, patients with an inflamed tumor stroma had a significant advantage in both overall and disease-specific survival (Fig. 6A,B). This was also observed for patients with a high T-cell infiltrate, who demonstrated significantly improved overall and disease-specific survival compared to those with a low T-cell infiltrate (Fig. 6C,D). As macrophages were ubiquitous to the post-treatment tumor microenvironment, there was no relationship between macrophage infiltration and survival (data not shown). Additionally, unlike our observations in pre-treatment specimens, there was no significant relationship between post-treatment neutrophil infiltration and overall or disease-specific survival (Fig. 6E,F).

## Discussion

Though clinical outcomes for anal SCC have improved significantly since the advent of the current standard of care chemoradiation regimen, survival is poor for patients who fail to show complete responses to first-line treatment [6]. At the present time, there is no clinically useful means to risk stratify patients for treatment failure. While T and N stages have been suggested to predict for poor clinical outcomes, data supporting their predictive value for treatment failure is limited [24, 25]. Similarly, though HPV-negative tumors tend to have worse outcomes and poorer therapeutic response rates than HPV-positive tumors, 90% of anal SCCs are HPV-positive, significantly limiting the utility of HPV as a predictive biomarker [26]. Hence, most intent-to-treat patients are treated similarly and without the use of any clinical or molecular biomarker.

To offer potential insight into patients unlikely to demonstrate complete therapeutic responses to the current standard of care, we documented a variety of objective clinical findings in a single center cohort of 42 patients with anal SCC and retrospectively related these to primary treatment outcome as well as overall and disease-specific survival. Consistent with previous reports [14], patients with a pre-treatment WBC count of ≥ 8 had particularly poor overall survival. However, we also determined that these patients have a comparative increase in the risk of treatment failure, as do those with an increased ANC. This was reflected in pre-treatment biopsies, where tumors with an inflamed, neutrophil dominant stroma also had extremely poor therapeutic responses, as well as reduced overall and disease specific survival.

Though these findings are interesting and potentially useful, it is important to note that this study has inherent limitations, particularly its small sample size. This is particularly true for histopathology experiments, which utilized leftover tissue specimens that were not available for all patients, particularly in the case of post-treatment specimens. Additionally, being a single center study, these findings may also be skewed by local demographics including a predominantly African American population and a disproportionately high rate of HIV infection. Hence, our findings should be validated in larger prospective, multi-site studies of more varied populations.

However, despite these limitations, our findings appear largely consistent with observations in other HPV-associated squamous cancers. For example, in both cervical and oropharyngeal SCC, increased tumor-infiltrating neutrophils are associated with poor survival [27, 28]. Similarly, in advanced head and neck SCC (HNSCC), tumors with dense neutrophil aggregates show particularly poor long-term survivability, presumptively due to tumor-permissive paracrine interactions between the HNSCC tumor cells and neutrophil infiltrate [29]. One recent study explored the relationship between tumor-associated neutrophils and outcomes in anal SCC using myeloperoxidase (MPO) as a marker, and found that neutrophils were not related to treatment outcome [16]. However, MPO is not specific to neutrophils, and is expressed by other myeloid cells including macrophages [30–32]. Hence, the role of neutrophils in anal SCC is poorly understood and warrants continued exploration using standardized methodology, particularly as neutrophils can seemingly contribute to the failure of both chemotherapy and radiation in other tumor types [33–35].

Importantly, neutrophils are emerging as a barrier to the therapeutic efficacy of immune checkpoint inhibitors (ICIs) [36–38]. Several recent trials have explored ICI-based immunotherapy for anal SCC. For example, the anti-PD-1 antibody Nivolumab has shown promise in patients with treatment-refractory metastatic anal SCC [39]. Similarly, the anti-PD-1 antibody Pembrolizumab has also shown durable anti-tumor activity in previously treated anal SCC, particularly for patients with PD-L1-expressing tumors [40–42]. Hence, it is important to determine whether enhanced neutrophil infiltration is a cause or consequence of treatment-refractory anal SCC, as well as whether neutrophil-depletion strategies will augment cytotoxic or immune therapies.

Finally, as a previous study has suggested that post-treatment peripheral leukocytosis may also predict for survival [16], we also evaluated the post-treatment CBC with differential as a potential prognostic biomarker. Though we observed uniform reductions in every major peripheral leukocyte subtype, we did not observe a statistically significant relationship between peripheral WBCs or ANC and therapy response, overall, or disease-specific survival. Interestingly, following treatment, patients with an inflamed tumor stroma had a significant survival advantage, particularly for those with a T-cell dominant stroma. These observations appear to suggest that chemoradiation may lead to extensive remodeling of the anal SCC tumor microenvironment, altering local immune cues. While our data suggests that the beneficial aspect of these events involves the apparent restoration of T-cell-mediated immune responses, it is important to note that this is still emerging in anal SCC.

For example, previous studies have found that patients with increased tumor-infiltrating lymphocytes have improved long-term survival independent of tumor stage [43]. Additional reports suggest that tumors demonstrating increased CD8 + T-cell infiltration have favorable clinical outcomes [44, 45], and that increased tumor-infiltrating lymphocytes are associated with lower rates of relapse [46]. However, another recent report also suggests that GranzymeB-positive cytotoxic T lymphocytes have a significant, negative impact on survival [47]. Hence, this warrants additional study in expanded cohorts that have also been controlled for additional factors such as age, HIV, and treatment status.

Regardless, our results suggest that routinely performed peripheral and tumor-infiltrating leukocyte subtyping may have potential utility in risk stratifying patients for treatment failure in anal SCC. Specifically, pre-treatment patients with a high WBC, ANC, and/or a neutrophil-dense tumor stroma may be less likely to achieve complete responses using the standard of care chemoradiation regimen, and may benefit from the addition of a subsequent line of therapy. Additionally, as enhanced post-treatment T-cell infiltration is a positive prognostic factor, the addition of ICIs such as Nivolumab or Pembrolizumab warrants consideration in patients who meet the above criteria as being unlikely to respond to the current standard of care.

## Conclusions

Our results suggest that routinely performed leukocyte subtyping may have utility in risk stratifying patients for treatment failure in anal SCC. Specifically, pre-treatment patients with a high WBC, ANC, and/or a neutrophil-dense tumor stroma may be less likely to achieve complete response using the standard of care chemoradiation regimen, and may benefit from the addition of a subsequent line of therapy.

## Supplementary Information

**Additional file 1:** **Figure S1. **Demographic information related to clinical outcomesin the anal SCC cohort. **Figure S2. **Natalsex does not relate to clinical outcomes in the anal SCC cohort. **Figure S3. **Clinical outcomes arrangedby tumor stage. **Figure S4. **Peripheralleukocyte counts arranged by demographics and tumor. **Figure S5.** Overall and disease-specific survival arranged bydemographic information. **Figure S6.**Pre-treatment anal SCCtumors with an inflamed stroma and/or increased neutrophil infiltrating areassociated with clinical outcomes. **Figure S7.** Overalland disease-specific survival arranged by demographic information. **Supplemental Dataset S1. **Pre-treatmentlab values, pre- and post-treatment histology results, and select demographicinformation for each individual patient. **TableS1. **Individualized clinical characteristics of anal SCC cohort. **Table S2. **Clinical characteristics ofanal SCC patient cohort arranged by clinical outcome. 

## Data Availability

Select non-mandated data has been uploaded as a supplementary dataset in accordance with BMC policy, excluding that with potentially identifiable patient information. For additional raw files or information please contact principe@uic.edu.

## Abbreviations

SCC: Squamous cell carcinoma
CBC: Complete blood count
WBC: White blood cells
ANC: Neutrophils
APR: Abdominoperineal resection
5-FU: 5-Fluorouracil
ICI: Immune checkpoint inhibition

## References

[1] Pessia B (2020). Squamous cell anal cancer: Management and therapeutic options. Ann Med Surg (Lond).

[2] van der Zee RP, Richel O, de Vries HJ, Prins JM (2013). The increasing incidence of anal cancer: can it be explained by trends in risk groups?. Neth J Med.

[3] Nigro ND (1983). Combined preoperative radiation and chemotherapy for squamous cell carcinoma of the anal canal. Cancer.

[4] Osborne MC, Maykel J, Johnson EK, Steele SR (2014). Anal squamous cell carcinoma: an evolution in disease and management. World J Gastroenterol.

[5] Glynne-Jones R (2017). Best time to assess complete clinical response after chemoradiotherapy in squamous cell carcinoma of the anus (ACT II): a post-hoc analysis of randomised controlled phase 3 trial. Lancet Oncol.

[6] Papaconstantinou HT, Bullard KM, Rothenberger DA, Madoff RD (2006). Salvage abdominoperineal resection after failed Nigro protocol: modest success, major morbidity. Colorectal Dis.

[7] Sterner A, Derwinger K, Staff C, Nilsson H, Angenete E (2019). Quality of life in patients treated for anal carcinoma-a systematic literature review. Int J Colorectal Dis.

[8] Lefevre JH (2012). Abdominoperineal resection for squamous cell anal carcinoma: survival and risk factors for recurrence. Ann Surg Oncol.

[9] Kim R (2014). Carboplatin and paclitaxel treatment is effective in advanced anal cancer. Oncology.

[10] Yhim HY (2011). The prognostic significance of tumor human papillomavirus status for patients with anal squamous cell carcinoma treated with combined chemoradiotherapy. Int J Cancer.

[11] Ravenda PS (2014). Prognostic value of human papillomavirus in anal squamous cell carcinoma. Cancer Chemother Pharmacol.

[12] Mai S (2015). Prognostic Relevance of HPV Infection and p16 Overexpression in Squamous Cell Anal Cancer. Int J Radiat Oncol Biol Phys.

[13] Rodel F (2015). Human papillomavirus DNA load and p16INK4a expression predict for local control in patients with anal squamous cell carcinoma treated with chemoradiotherapy. Int J Cancer.

[14] Glynne-Jones R (2013). Prognostic factors for recurrence and survival in anal cancer: generating hypotheses from the mature outcomes of the first United Kingdom Coordinating Committee on Cancer Research Anal Cancer Trial (ACT I). Cancer.

[15] Banerjee R (2013). The prognostic significance of pretreatment leukocytosis in patients with anal cancer treated with radical chemoradiotherapy or radiotherapy. Dis Colon Rectum.

[16] Martin D (2017). Peripheral Leukocytosis Is Inversely Correlated with Intratumoral CD8+ T-Cell Infiltration and Associated with Worse Outcome after Chemoradiotherapy in Anal Cancer. Front Immunol.

[17] Schernberg A (2017). Leukocytosis and neutrophilia predicts outcome in anal cancer. Radiother Oncol.

[18] Schernberg A (2017). External validation of leukocytosis and neutrophilia as a prognostic marker in anal carcinoma treated with definitive chemoradiation. Radiother Oncol.

[19] Principe DR (2020). Long-Term Gemcitabine Treatment Reshapes the Pancreatic Tumor Microenvironment and Sensitizes Murine Carcinoma to Combination Immunotherapy. Cancer Res.

[20] Principe DR (2019). TGFbeta Blockade Augments PD-1 Inhibition to Promote T-Cell-Mediated Regression of Pancreatic Cancer. Mol Cancer Ther.

[21] Principe DR (2022). Loss of SMAD4 Is Associated With Poor Tumor Immunogenicity and Reduced PD-L1 Expression in Pancreatic Cancer. Front Oncol.

[22] Principe DR (2022). XP-524 is a dual-BET/EP300 inhibitor that represses oncogenic KRAS and potentiates immune checkpoint inhibition in pancreatic cancer. Proc Natl Acad Sci U S A.

[23] Principe DR, Kamath SD, Korc M, Munshi HG (2022). The immune modifying effects of chemotherapy and advances in chemo-immunotherapy. Pharmacol Ther.

[24] Gunderson LL (2013). Anal carcinoma: impact of TN category of disease on survival, disease relapse, and colostomy failure in US Gastrointestinal Intergroup RTOG 98–11 phase 3 trial. Int J Radiat Oncol Biol Phys.

[25] Das P, Crane CH, Eng C, Ajani JA (2008). Prognostic factors for squamous cell cancer of the anal canal. Gastrointest Cancer Res.

[26] Meulendijks D (2015). HPV-negative squamous cell carcinoma of the anal canal is unresponsive to standard treatment and frequently carries disruptive mutations in TP53. Br J Cancer.

[27] Matsumoto Y (2017). The significance of tumor-associated neutrophil density in uterine cervical cancer treated with definitive radiotherapy. Gynecol Oncol.

[28] Li C (2019). Neutrophils infiltration and its correlation with human papillomavirus status in the oral squamous cell carcinoma. Cancer Manag Res.

[29] Trellakis S (2011). Polymorphonuclear granulocytes in human head and neck cancer: enhanced inflammatory activity, modulation by cancer cells and expansion in advanced disease. Int J Cancer.

[30] Sugiyama S (2001). Macrophage myeloperoxidase regulation by granulocyte macrophage colony-stimulating factor in human atherosclerosis and implications in acute coronary syndromes. Am J Pathol.

[31] McMillen TS, Heinecke JW, LeBoeuf RC (2005). Expression of human myeloperoxidase by macrophages promotes atherosclerosis in mice. Circulation.

[32] Shaeib F (2016). The Impact of Myeloperoxidase and Activated Macrophages on Metaphase II Mouse Oocyte Quality. PLoS ONE.

[33] Nywening TM (2018). Targeting both tumour-associated CXCR2(+) neutrophils and CCR2(+) macrophages disrupts myeloid recruitment and improves chemotherapeutic responses in pancreatic ductal adenocarcinoma. Gut.

[34] Wisdom AJ (2019). Neutrophils promote tumor resistance to radiation therapy. Proc Natl Acad Sci U S A.

[35] Shinde-Jadhav S (2021). Role of neutrophil extracellular traps in radiation resistance of invasive bladder cancer. Nat Commun.

[36] Wang PF (2020). Neutrophil depletion enhances the therapeutic effect of PD-1 antibody on glioma. Aging (Albany NY).

[37] Zhang Y (2020). Interleukin-17-induced neutrophil extracellular traps mediate resistance to checkpoint blockade in pancreatic cancer. J Exp Med.

[38] Nielsen SR (2021). Suppression of tumor-associated neutrophils by lorlatinib attenuates pancreatic cancer growth and improves treatment with immune checkpoint blockade. Nat Commun.

[39] Morris VK (2017). Nivolumab for previously treated unresectable metastatic anal cancer (NCI9673): a multicentre, single-arm, phase 2 study. Lancet Oncol.

[40] Ott PA (2017). Safety and antitumor activity of the anti-PD-1 antibody pembrolizumab in patients with recurrent carcinoma of the anal canal. Ann Oncol.

[41] Marabelle A (2020). Pembrolizumab for advanced anal squamous cell carcinoma (ASCC): Results from the multicohort, phase II KEYNOTE-158 study. J Clin Oncol.

[42] Marabelle A (2020). Pembrolizumab for previously treated advanced anal squamous cell carcinoma: Pooled results from the KEYNOTE-028 and KEYNOTE-158 studies. J Clin Oncol.

[43] Rubio CA (2008). The clinical significance of massive intratumoral lymphocytosis in squamous cell carcinoma of the anus. Int J Clin Exp Pathol.

[44] Balermpas P (2017). Human papilloma virus load and PD-1/PD-L1, CD8(+) and FOXP3 in anal cancer patients treated with chemoradiotherapy: Rationale for immunotherapy. Oncoimmunology.

[45] Hu WH (2015). Tumor-infiltrating CD8(+) T lymphocytes associated with clinical outcome in anal squamous cell carcinoma. J Surg Oncol.

[46] Gilbert DC (2016). Tumour-infiltrating lymphocyte scores effectively stratify outcomes over and above p16 post chemo-radiotherapy in anal cancer. Br J Cancer.

[47] Grabenbauer GG, Lahmer G, Distel L, Niedobitek G (2006). Tumor-infiltrating cytotoxic T cells but not regulatory T cells predict outcome in anal squamous cell carcinoma. Clin Cancer Res.

