# Supplementary material for: Leukocyte subtyping predicts for treatment failure and poor survival in anal squamous cell carcinoma
Source: BMC Cancer. 2022 Jun 24;22:697. doi: 10.1186/s12885-022-09742-7 (PMC9229146; doi:10.1186/s12885-022-09742-7)
Supplement: Supplementary file 1 — Additional file 1: Figure S1. Demographic information related to clinical outcomesin the anal SCC cohort. Figure S2. Natalsex does not relate to clinical outcomes in the anal SCC cohort. Figure S3. Clinical outcomes arrangedby tumor stage. Figure S4. Peripheralleukocyte counts arranged by demographics and tumor. Figure S5. Overall and disease-specific survival arranged bydemographic information. Figure S6.Pre-treatment anal SCCtumors with an inflamed stroma and/or increased neutrophil infiltrating areassociated with clinical outcomes. Figure S7. Overalland disease-specific survival arranged by demographic information. Supplemental Dataset S1. Pre-treatmentlab values, pre- and post-treatment histology results, and select demographicinformation for each individual patient. TableS1. Individualized clinical characteristics of anal SCC cohort. Table S2. Clinical characteristics ofanal SCC patient cohort arranged by clinical outcome. [file 12885_2022_9742_MOESM1_ESM.zip › 12885_2022_9742_MOESM1_ESM/Anal SCC Supplement Third Submission .docx]

**Supplement: Leukocyte Subtyping Predicts for Treatment Failure and Poor Survival in Anal Squamous Cell Carcinoma**

Daniel R. Principe^1,2^, Jose L. Cataneo^2^, Kaytlin E. Timbers^2^, Regina M. Koch^2^, Klara Valyi-Nagy^3^, Anders Mellgren^2^, Ajay Rana^2,4^, and Gerald Gantt^2^

^1^Medical Scientist Training Program, University of Illinois College of Medicine, Chicago, IL; ^2^Department of Surgery, University of Illinois at Chicago, Chicago, IL; ^3^Department of Pathology, University of Illinois at Chicago, Chicago, IL; ^4^Jesse Brown VA Medical Center, Chicago, IL.

Short Title: Leukocyte subtyping predicts outcomes in anal cancer

Pages 5

Abstract 249

Tables 1

Figures 7

Words 567

^#^Correspondence to:

Gerald A. Gantt Jr

Department of Surgery

Division of Colorectal Surgery

College of Medicine

The University of Illinois at Chicago

840 S. Wood Street

Suite 509 Clinical Sciences Building

Chicago, IL 60612

Tel: (312) 996-2061

Email: [ggantt2@uic.edu](mailto:ggantt2@uic.edu)

**SUPPLEMENTAL FIGURE LEGENDS**

**Figure S1. Demographic information related to clinical outcomes in the anal SCC cohort**

**(A)** Basic demographic information in the form of natal sex, race, and HIV status for the 42 patients with anal SCC enrolled in this study. **(B)** Based on the described criteria, the percent of patients demonstrating complete responses to therapy (Respond), or failing to show complete responses in the form of persistent or recurrent disease (Fail). **(C,D)** The percent overall survival (OS) or disease-specific survival (DSS) for all patients in the anal SCC cohort. **(E,F)** OS or DSS for patients arranged by primary therapy outcome. **(G,H)** Primary therapy outcome arranged by either HIV status or race (AA = African America, AA = Caucasian). **(I,J)** OS arranged by either HIV status or race. **(K,L)** DSS arranged by either HIV status or race.

**Figure S2. Natal sex does not relate to clinical outcomes in the anal SCC cohort**

**(A)** Primary therapy outcome, **(B)** overall survival (OS), and **(C)** disease-specific survival arranged by natal sex.

**Figure S3. Clinical outcomes arranged by tumor stage**

**(A)** Primary therapy outcome, **(B)** overall survival (OS), and **(C)** disease-specific survival arranged by tumor stage. **(D,E)** **(A)** Kaplan-Meier curve indicating months of overall or disease-specific survival for post-treatment anal SCC patients arranged by tumor stage.

**Figure S4. Peripheral leukocyte counts arranged by demographics and tumor stage**

Peripheral blood specimens were collected from 42 chemo-naïve patients with anal SCC, and subjected to routine complete blood count (CBC) with differential. Using these data, the number of peripheral **(A)** white blood cells (WBCs) **(B)** absolute neutrophil count, or **(C)** basophils were related to natal sex, race (AA = African America, AA = Caucasian), HIV status, and/or tumor stage.

**Figure S5.** **Overall and disease-specific survival arranged by demographic information**

Kaplan-Meier curve indicating months of overall or disease-specific survival for post-treatment anal SCC patients arranged by **(A,B)** natal sex, **(C,D)** race (AA = African America, AA = Caucasian), **(E,F)** HIV status, or **(G,H)** an pre-treatment absolute neutrophil count (ANC) of <5 or an ANC of ≥5.

**Figure S6. Pre-treatment anal SCC tumors with an inflamed stroma and/or increased neutrophil infiltrating are associated with clinical outcomes**

Pre-treatment excisional biopsies from 27 chemo-naïve anal SCC patients were sectioned, stained, and quantified as described. Primary therapy outcome, overall survival (OS), and/or disease-specific survival arranged by **(A)** p16INK4a (P16) status, **(B)** an inflamed or uninflamed tumor stroma, or the degree of **(C)** T-cell, **(D)** macrophage, or **(E)** neutrophil infiltration.

**Figure S7.** **Overall and disease-specific survival arranged by demographic information**

Peripheral blood specimens were collected from 40 patients with anal SCC after completing chemo-radiation. **(A)** Using these data, the number of peripheral white blood cells (WBCs), absolute neutrophil count (ANC), lymphocytes, CD4+ T-cells for HIV patients, monocytes, and eosinophils were compared to pre-treatment values. Post-treatment peripheral blood counts were next related to **(B)**, primary therapy outcome **(C)**, overall survival (OS), or **(D)** disease-specific survival (DSS).

**Supplemental Dataset S1. Pre-treatment lab values, pre- and post-treatment histology results, and select demographic information for each individual patient.**

**SUPPLEMENTARY TABLES**

| **Patient**  **ID** | **Age at**  **Diagnosis** | **Natal Sex** | **Race** | **Stage at Diagnosis** | **HIV** | **HAART** | **Crohn’s**  **Disease** | **History of Transplant** | **History of Cervical**  **Cancer** | **Smoking**  **Status** |
| --- | --- | --- | --- | --- | --- | --- | --- | --- | --- | --- |
| 1 | 57 | Female | White | 1 | No | - | No | No | No | Never |
| 2 | 65 | Female | Other | 3 | No | - | No | No | Yes | Former |
| 3 | 72 | Male | White | 2 | No | - | No | No | No | Current |
| 4 | 42 | Female | Black | 3 | No | - | No | No | No | Current |
| 5 | 68 | Female | Black | 1 | No | - | No | No | No | Former |
| 6 | 63 | Male | Other | 3 | No | - | Yes | No | No | Former |
| 7 | 63 | Male | White | 2 | Yes | Efavirenz, Emtricitabine, Tenofovir | No | No | No | Current |
| 8 | 61 | Male | White | 3 | Yes | Bictegravir, Emtricitabine, Tenofovir | No | No | No | Never |
| 9 | 57 | Male | Black | 3 | No | - | No | No | No | Never |
| 10 | 44 | Female | Black | 1 | No | - | No | No | No | Former |
| 11 | 73 | Male | White | - | No | - | No | No | No | Former |
| 12 | 55 | Male | White | 4 | Yes | Emtricitabine, Tenofovir | No | No | No | Current |
| 13 | 59 | Male | Black | 3 | Yes | Emtricitabine, Rilpivirine,  Tenofovir | No | No | No | Current |
| 14 | 61 | Female | Black | 3 | No | - | No | No | No | Current |
| 15 | 62 | Male | Black | 3 | Yes | - | No | No | No | Former |
| 16 | 42 | Male | White | 2 | Yes | Emtricitabine, Tenofovir | No | No | No | Current |
| 17 | 54 | Male | Other | 3 | No | - | No | No | No | Never |
| 18 | 39 | Male | Black | 3 | Yes | Efavirenz, Emtricitabine, Tenofovir | No | No | No | Never |
| 19 | 66 | Female | Black | 3 | No | - | No | No | No | Never |
| 20 | 56 | Male | Other | 3 | Yes | Emtricitabine, Tenofovir | No | No | No | Current |
| 21 | 50 | Female | White | 3 | No | - | No | No | No | Current |
| 22 | 50 | Male | Black | - | Yes | Emtricitabine, Tenofovir | No | No | No | Current |
| 23 | 55 | Male | Other | 3 | Yes | Lamivudine | No | No | No | Current |
| 24 | 49 | Female | Black | 3 | No | Azathioprine | No | No | Yes | Current |
| 25 | 43 | Male | Black | 1 | Yes | Abacavir, Lamivudine | No | No | No | Current |
| 26 | 60 | Female | Black | 2 | No | Tacrolimus | No | Yes | No | Never |
| 27 | 54 | Male | Black | 2 | No | - | No | No | No | Current |
| 28 | 39 | Female | Black | 1 | Yes | Efavirenz, Emtricitabine, Tenofovir | No | No | No | Current |
| 29 | 65 | Male | Black | 4 | No | - | No | No | No | Former |
| 30 | 38 | Female | Black | 3 | No | - | No | No | No | Current |
| 31 | 51 | Male | Black | 2 | Yes | Emtricitabine, Tenofovir | No | No | No | Current |
| 32 | 44 | Male | White | 1 | Yes | HAART Unknown | No | No | No | Never |
| 33 | 55 | Female | White | 3 | No | - | No | No | No | Current |
| 34 | 56 | Female | White | 2 | No | - | No | No | No | Current |
| 35 | 33 | Male | Black | 1 | Yes | Efavirenz, Emtricitabine, Tenofovir | No | No | No | Former |
| 36 | 36 | Female | Black | 1 | Yes | Abacavir,  Lamivudine | No | No | No | Current |
| 37 | 46 | Female | White | 1 | Yes | HAART Unknown | No | No | No | Current |
| 38 | 52 | Male | Black | 1 | Yes | HAART Unknown | No | No | No | Former |
| 39 | 38 | Male | Black | 3 | Yes | Lopinavir, Ritonavir | No | No | No | Never |
| 40 | 56 | Male | Black | 2 | Yes | Lamivudine, Zidovudine | No | No | No | Current |
| 41 | 47 | Male | White | 3 | No | - | No | No | No | Former |
| 42 | 37 | Male | Black | 1 | Yes | Efavirenz, Didanosine, Lamivudine | No | No | No | Current |

**Table S1. Individualized clinical characteristics of anal SCC cohort**

| **Category** | **Number of Patients** | **Percent of Total** |
| --- | --- | --- |
|  |  |  |
| **Natal Sex** |  |  |
| Male | 26 | 38.1% |
| Female | 16 | 61.9% |
|  |  |  |
| **Race** |  |  |
| Black/AA | 24 | 57.1% |
| White/CA | 13 | 31% |
| Other | 5 | 11.9% |
|  |  |  |
| **Stage** |  |  |
| 1 | 11 | 26.2% |
| 2 | 8 | 19% |
| 3 | 19 | 45.2% |
| 4 | 2 | 4.8% |
| Not Reported | 2 | 4.8% |
|  |  |  |
| **HIV** |  |  |
| Positive | 21 | 50% |
| Negative | 21 | 50% |
|  |  |  |
| **Primary Therapy Outcome** |  |  |
| Respond | 34 | 81% |
| Fail | 8 | 19% |
|  |  |  |
| **Combined Vital Status** |  |  |
| Alive | 28 | 66.7% |
| Dead | 14 | 33.3% |
|  |  |  |
| **Disease Specific Vital Status** |  |  |
| Alive | 28 | 75.7% |
| Dead | 9 | 24.3% |
|  |  |  |
|  |  |  |

**Table S2. Clinical characteristics of anal SCC patient cohort arranged by clinical outcome**
