# Supplementary figures and images for: Leukocyte subtyping predicts for treatment failure and poor survival in anal squamous cell carcinoma
Source: BMC Cancer. 2022 Jun 24;22:697. doi: 10.1186/s12885-022-09742-7 (PMC9229146; doi:10.1186/s12885-022-09742-7)

**A**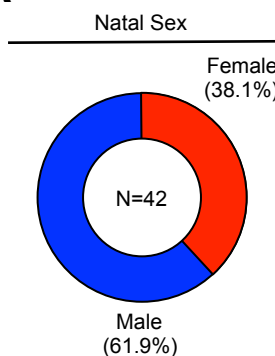**B**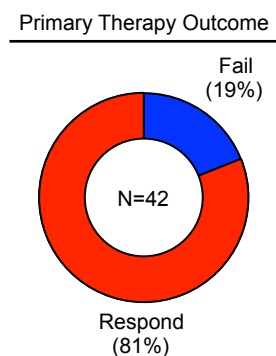**C**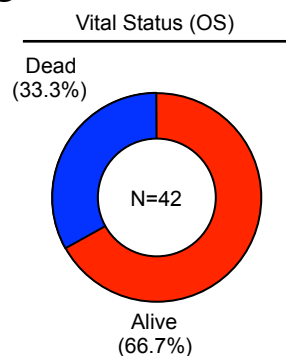**D**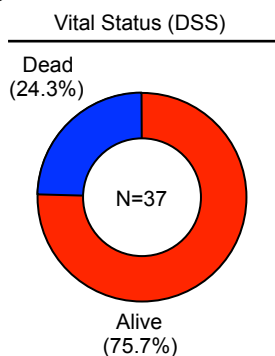**E**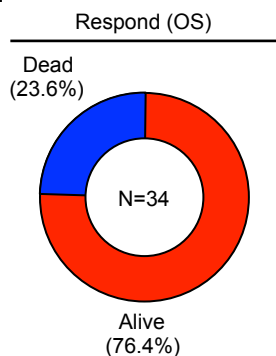**F**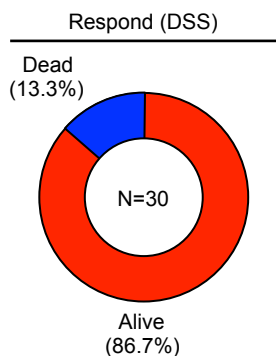**G**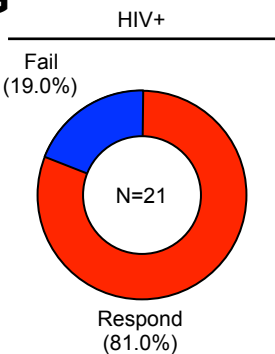**H**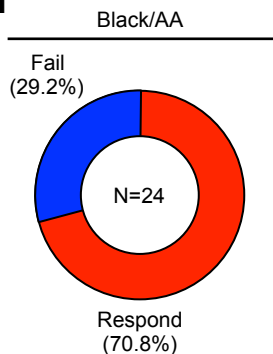**I**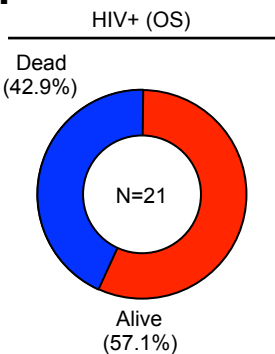**J**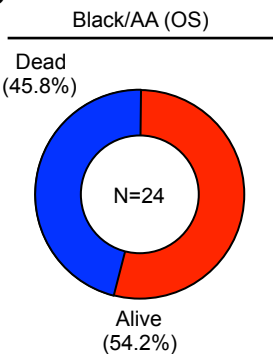**K**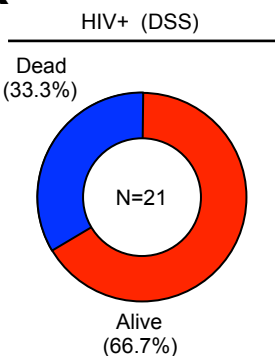**L**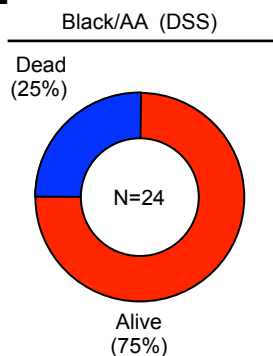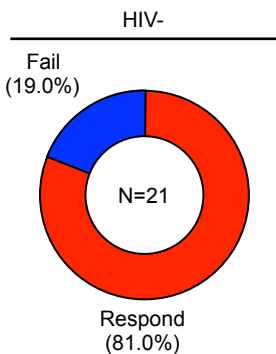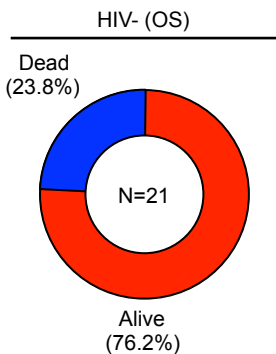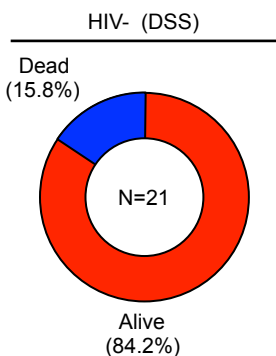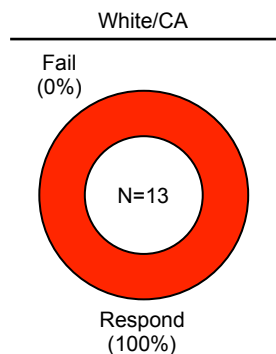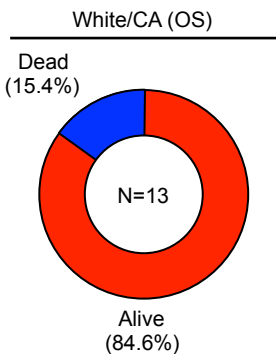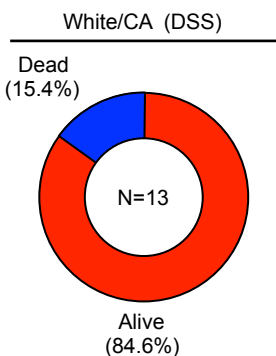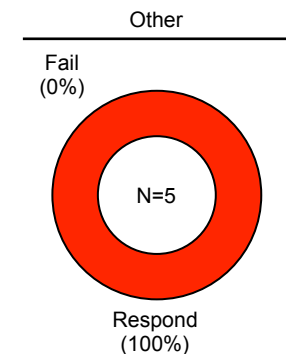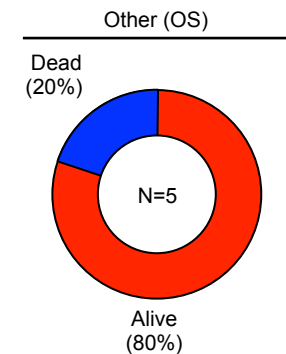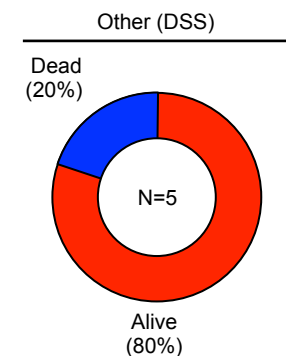

Supplement: Supplementary file 1 — Additional file 1: Figure S1. Demographic information related to clinical outcomesin the anal SCC cohort. Figure S2. Natalsex does not relate to clinical outcomes in the anal SCC cohort. Figure S3. Clinical outcomes arrangedby tumor stage. Figure S4. Peripheralleukocyte counts arranged by demographics and tumor. Figure S5. Overall and disease-specific survival arranged bydemographic information. Figure S6.Pre-treatment anal SCCtumors with an inflamed stroma and/or increased neutrophil infiltrating areassociated with clinical outcomes. Figure S7. Overalland disease-specific survival arranged by demographic information. Supplemental Dataset S1. Pre-treatmentlab values, pre- and post-treatment histology results, and select demographicinformation for each individual patient. TableS1. Individualized clinical characteristics of anal SCC cohort. Table S2. Clinical characteristics ofanal SCC patient cohort arranged by clinical outcome. [file 12885_2022_9742_MOESM1_ESM.zip › 12885_2022_9742_MOESM1_ESM/Figure S1.pdf]

**A**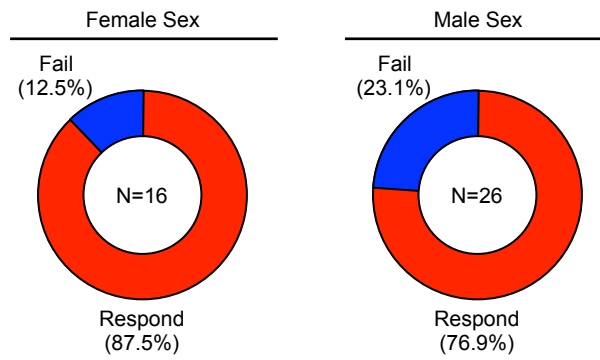**B**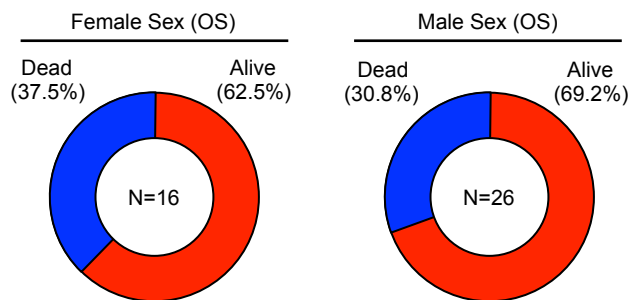**C**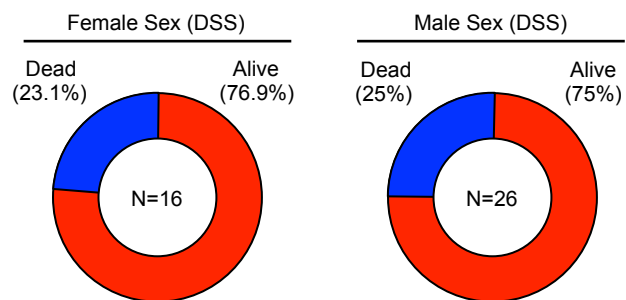

Supplement: Supplementary file 1 — Additional file 1: Figure S1. Demographic information related to clinical outcomesin the anal SCC cohort. Figure S2. Natalsex does not relate to clinical outcomes in the anal SCC cohort. Figure S3. Clinical outcomes arrangedby tumor stage. Figure S4. Peripheralleukocyte counts arranged by demographics and tumor. Figure S5. Overall and disease-specific survival arranged bydemographic information. Figure S6.Pre-treatment anal SCCtumors with an inflamed stroma and/or increased neutrophil infiltrating areassociated with clinical outcomes. Figure S7. Overalland disease-specific survival arranged by demographic information. Supplemental Dataset S1. Pre-treatmentlab values, pre- and post-treatment histology results, and select demographicinformation for each individual patient. TableS1. Individualized clinical characteristics of anal SCC cohort. Table S2. Clinical characteristics ofanal SCC patient cohort arranged by clinical outcome. [file 12885_2022_9742_MOESM1_ESM.zip › 12885_2022_9742_MOESM1_ESM/Figure S2.pdf]

**A**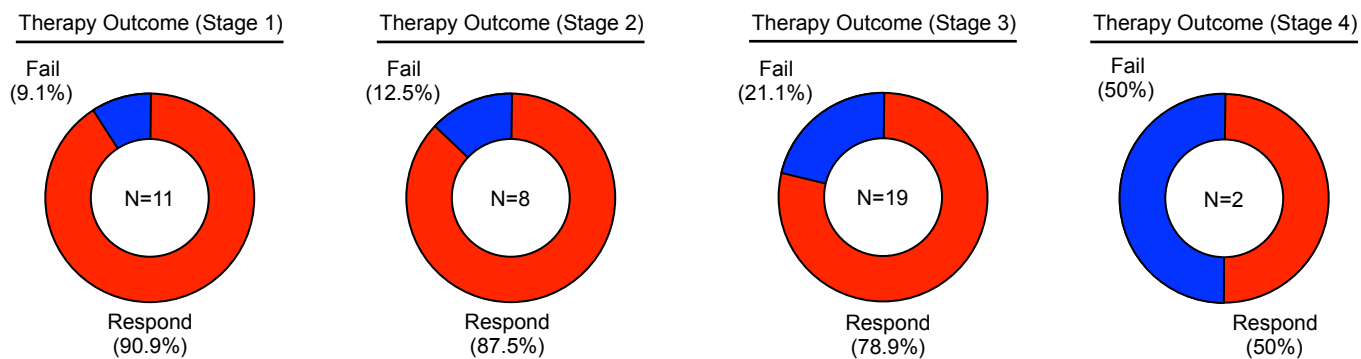**B**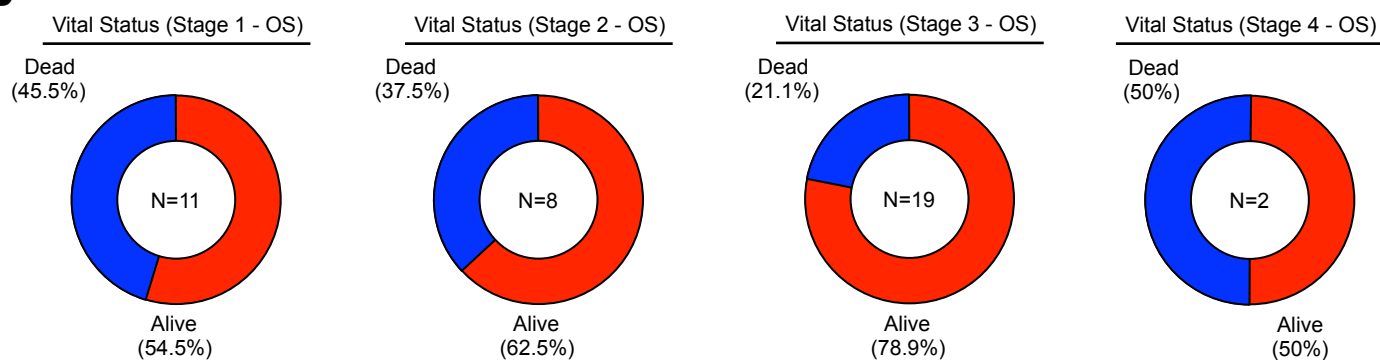**C**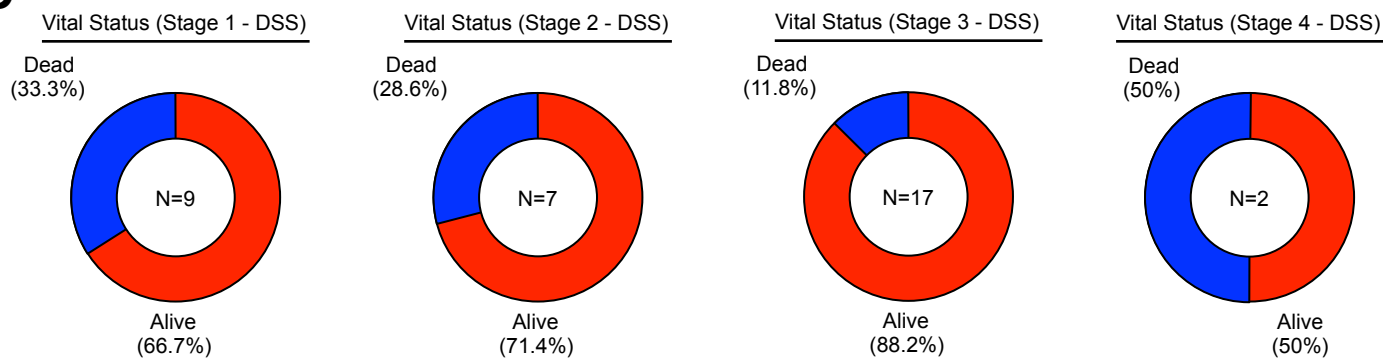**D**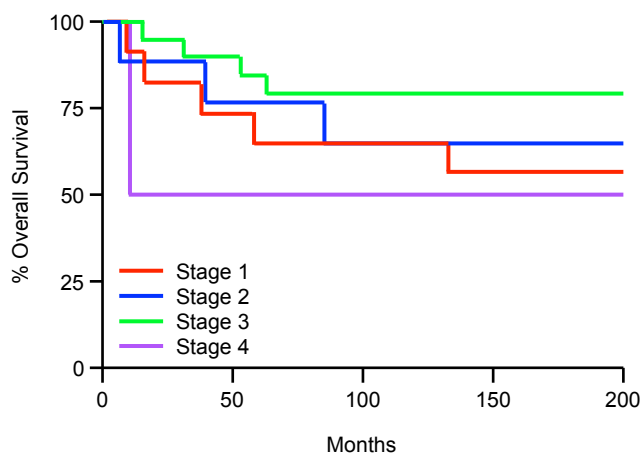**E**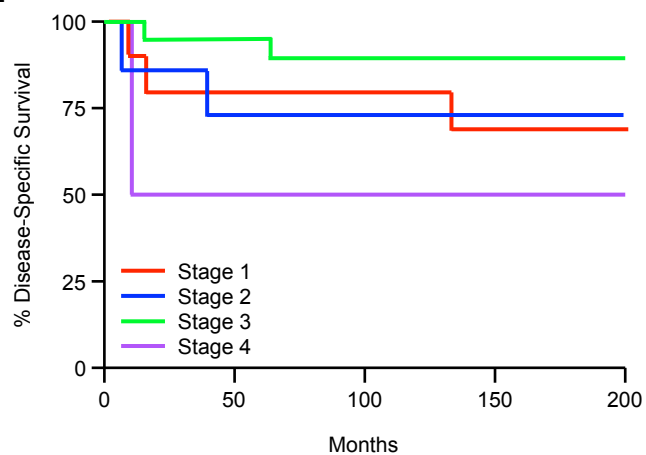

Supplement: Supplementary file 1 — Additional file 1: Figure S1. Demographic information related to clinical outcomesin the anal SCC cohort. Figure S2. Natalsex does not relate to clinical outcomes in the anal SCC cohort. Figure S3. Clinical outcomes arrangedby tumor stage. Figure S4. Peripheralleukocyte counts arranged by demographics and tumor. Figure S5. Overall and disease-specific survival arranged bydemographic information. Figure S6.Pre-treatment anal SCCtumors with an inflamed stroma and/or increased neutrophil infiltrating areassociated with clinical outcomes. Figure S7. Overalland disease-specific survival arranged by demographic information. Supplemental Dataset S1. Pre-treatmentlab values, pre- and post-treatment histology results, and select demographicinformation for each individual patient. TableS1. Individualized clinical characteristics of anal SCC cohort. Table S2. Clinical characteristics ofanal SCC patient cohort arranged by clinical outcome. [file 12885_2022_9742_MOESM1_ESM.zip › 12885_2022_9742_MOESM1_ESM/Figure S3.pdf]

**A**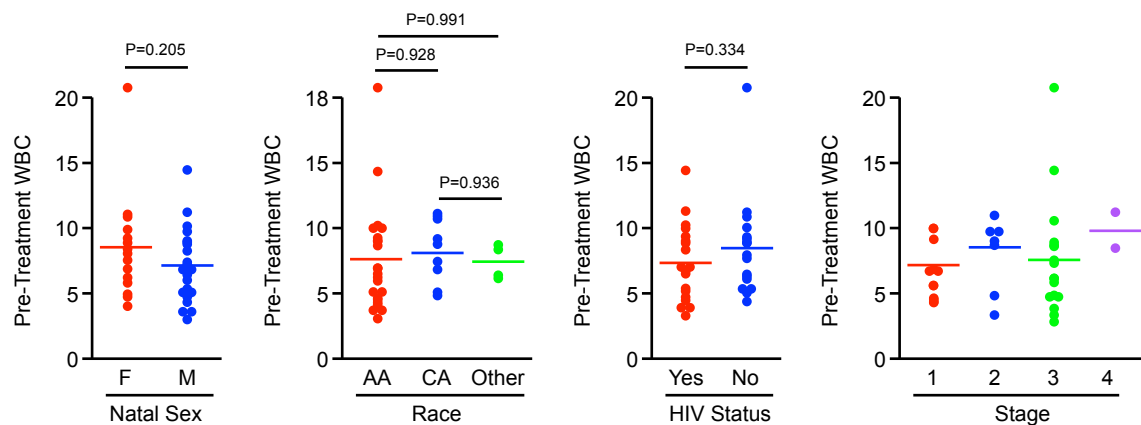**B**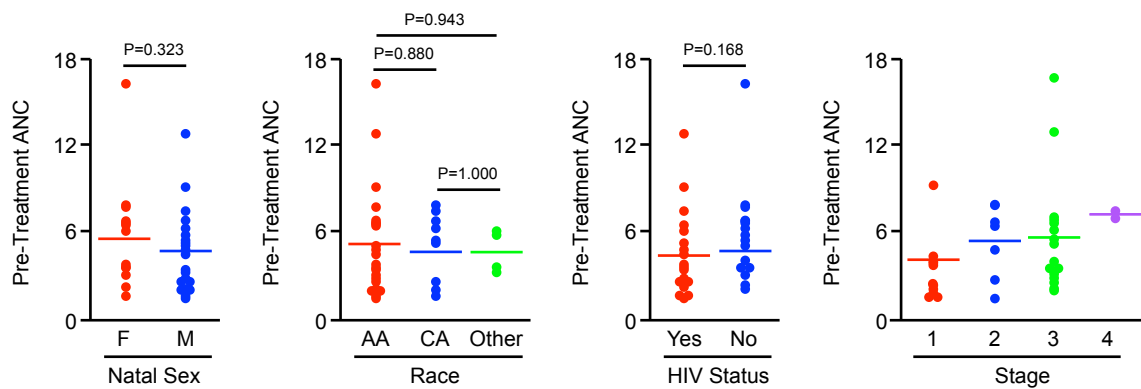**C**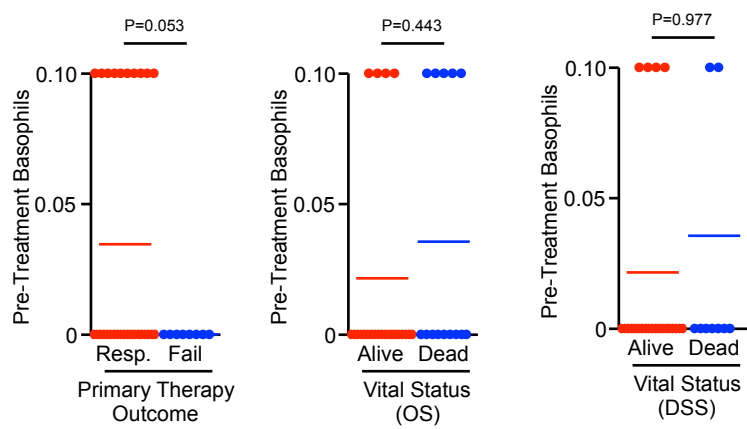

Supplement: Supplementary file 1 — Additional file 1: Figure S1. Demographic information related to clinical outcomesin the anal SCC cohort. Figure S2. Natalsex does not relate to clinical outcomes in the anal SCC cohort. Figure S3. Clinical outcomes arrangedby tumor stage. Figure S4. Peripheralleukocyte counts arranged by demographics and tumor. Figure S5. Overall and disease-specific survival arranged bydemographic information. Figure S6.Pre-treatment anal SCCtumors with an inflamed stroma and/or increased neutrophil infiltrating areassociated with clinical outcomes. Figure S7. Overalland disease-specific survival arranged by demographic information. Supplemental Dataset S1. Pre-treatmentlab values, pre- and post-treatment histology results, and select demographicinformation for each individual patient. TableS1. Individualized clinical characteristics of anal SCC cohort. Table S2. Clinical characteristics ofanal SCC patient cohort arranged by clinical outcome. [file 12885_2022_9742_MOESM1_ESM.zip › 12885_2022_9742_MOESM1_ESM/Figure S4.pdf]

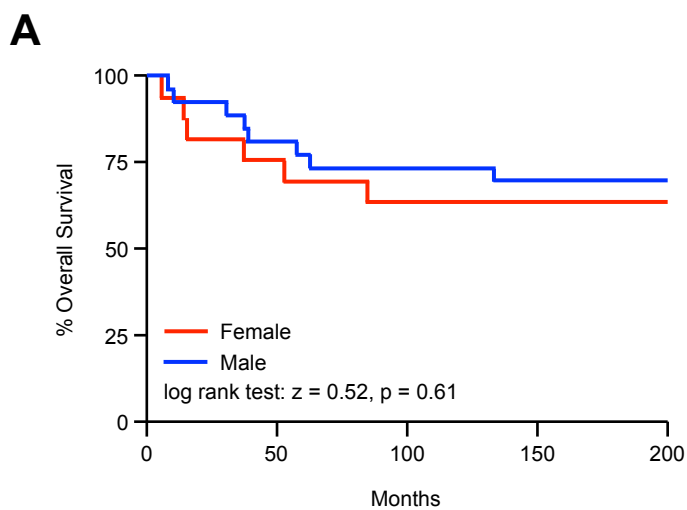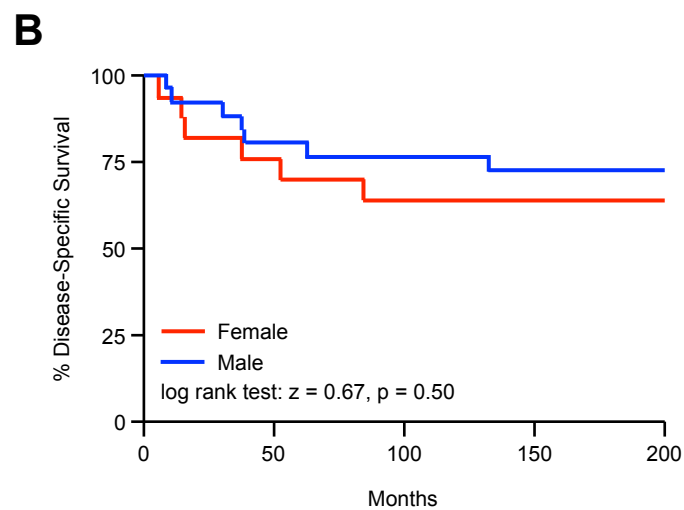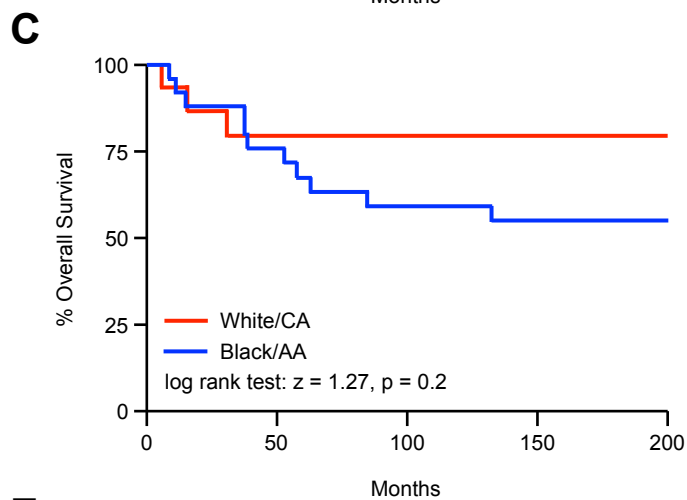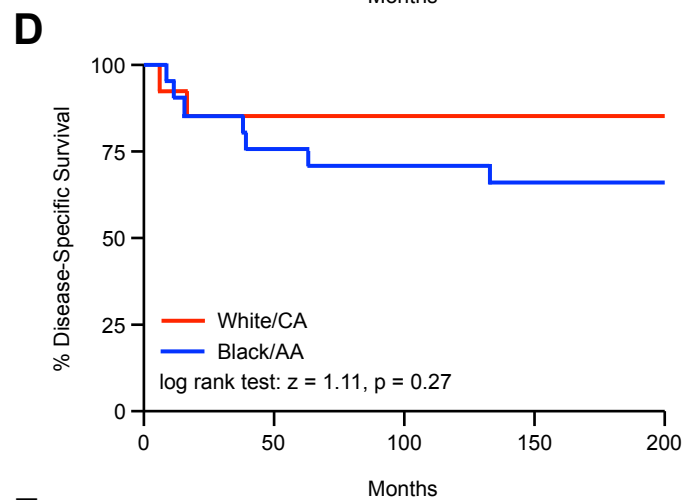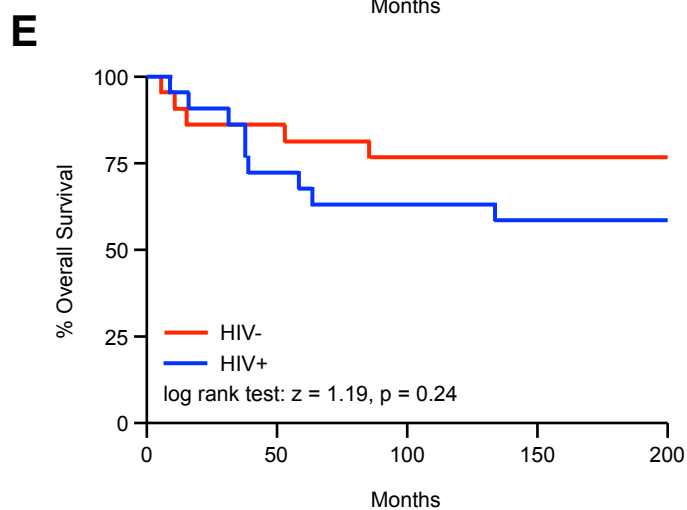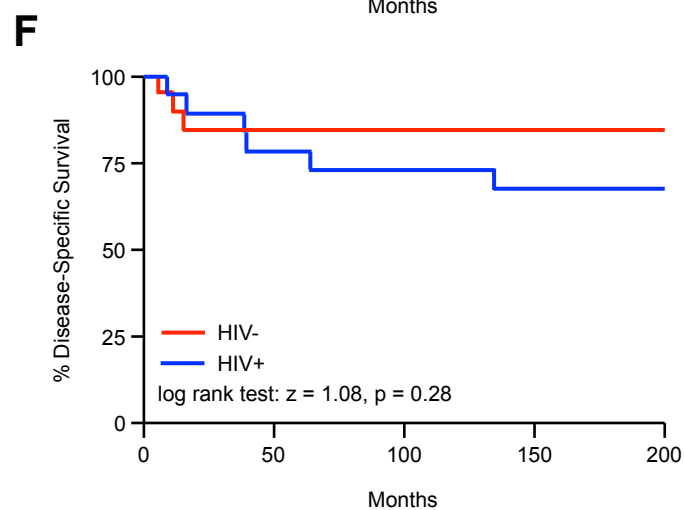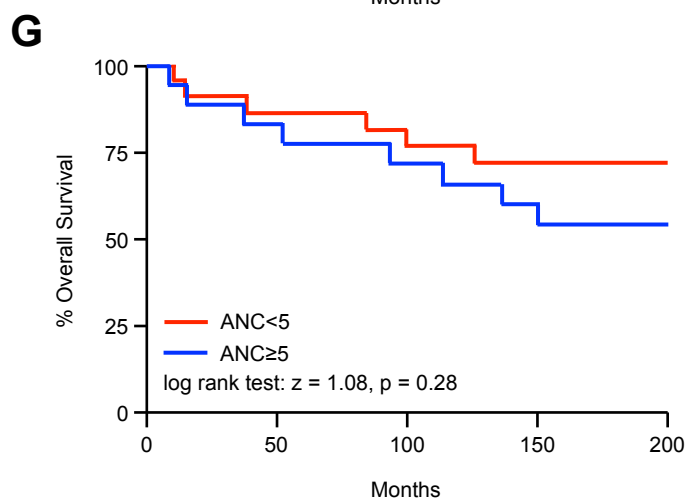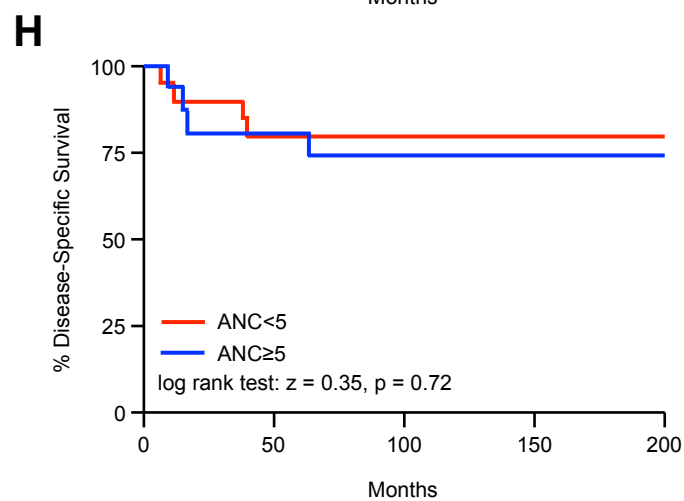

Supplement: Supplementary file 1 — Additional file 1: Figure S1. Demographic information related to clinical outcomesin the anal SCC cohort. Figure S2. Natalsex does not relate to clinical outcomes in the anal SCC cohort. Figure S3. Clinical outcomes arrangedby tumor stage. Figure S4. Peripheralleukocyte counts arranged by demographics and tumor. Figure S5. Overall and disease-specific survival arranged bydemographic information. Figure S6.Pre-treatment anal SCCtumors with an inflamed stroma and/or increased neutrophil infiltrating areassociated with clinical outcomes. Figure S7. Overalland disease-specific survival arranged by demographic information. Supplemental Dataset S1. Pre-treatmentlab values, pre- and post-treatment histology results, and select demographicinformation for each individual patient. TableS1. Individualized clinical characteristics of anal SCC cohort. Table S2. Clinical characteristics ofanal SCC patient cohort arranged by clinical outcome. [file 12885_2022_9742_MOESM1_ESM.zip › 12885_2022_9742_MOESM1_ESM/Figure S5.pdf]

**A**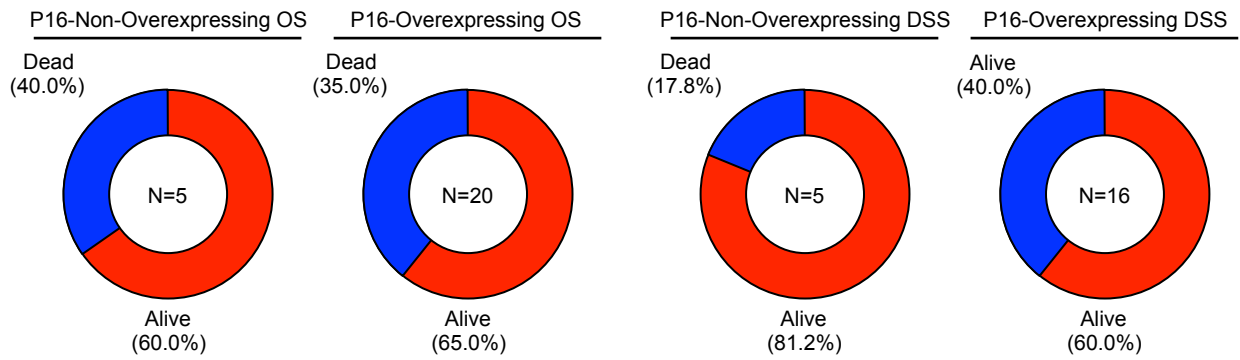**B**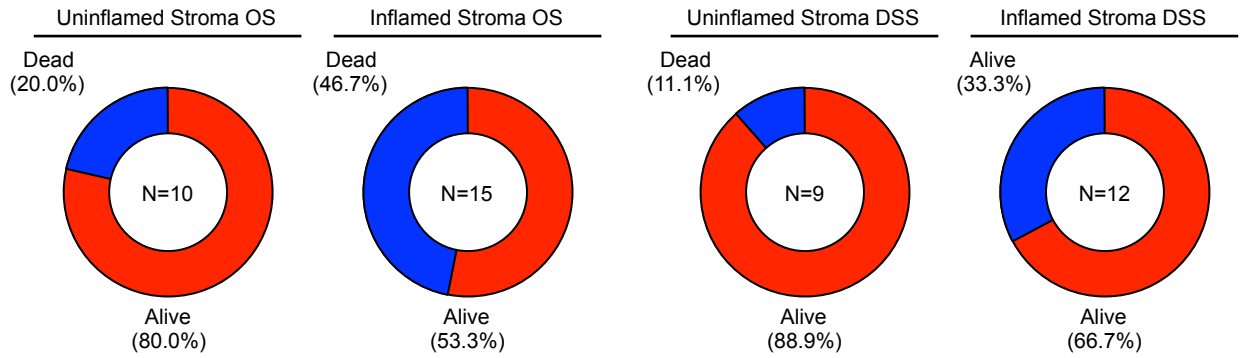**C**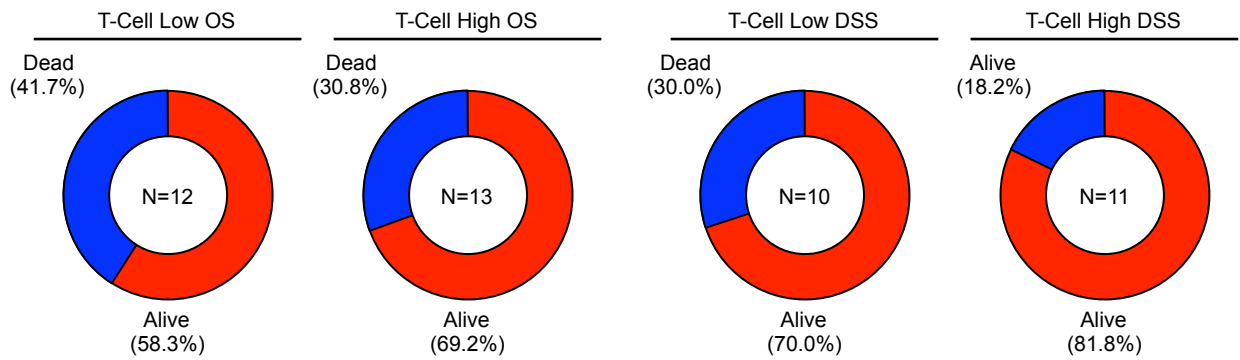**D**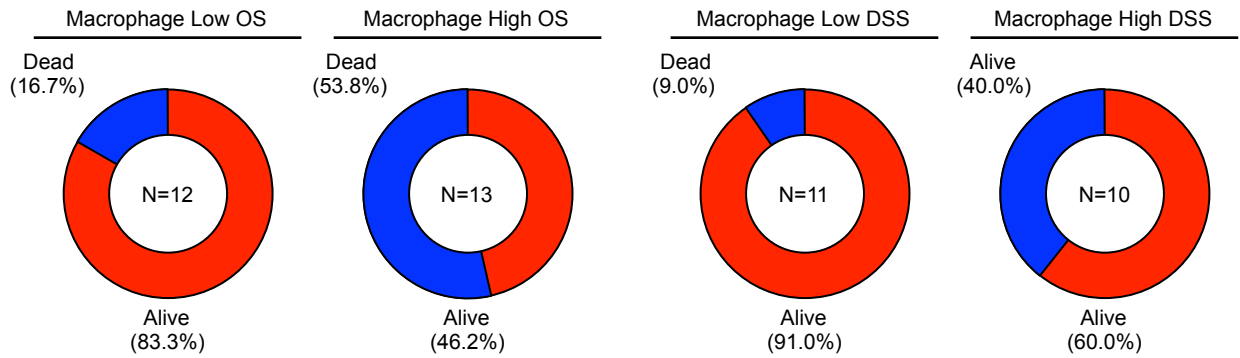**E**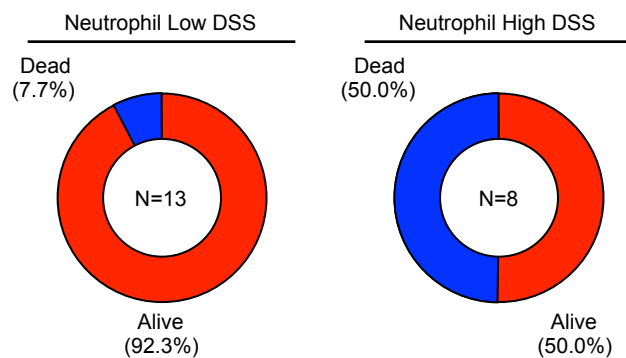

Supplement: Supplementary file 1 — Additional file 1: Figure S1. Demographic information related to clinical outcomesin the anal SCC cohort. Figure S2. Natalsex does not relate to clinical outcomes in the anal SCC cohort. Figure S3. Clinical outcomes arrangedby tumor stage. Figure S4. Peripheralleukocyte counts arranged by demographics and tumor. Figure S5. Overall and disease-specific survival arranged bydemographic information. Figure S6.Pre-treatment anal SCCtumors with an inflamed stroma and/or increased neutrophil infiltrating areassociated with clinical outcomes. Figure S7. Overalland disease-specific survival arranged by demographic information. Supplemental Dataset S1. Pre-treatmentlab values, pre- and post-treatment histology results, and select demographicinformation for each individual patient. TableS1. Individualized clinical characteristics of anal SCC cohort. Table S2. Clinical characteristics ofanal SCC patient cohort arranged by clinical outcome. [file 12885_2022_9742_MOESM1_ESM.zip › 12885_2022_9742_MOESM1_ESM/Figure S6.pdf]

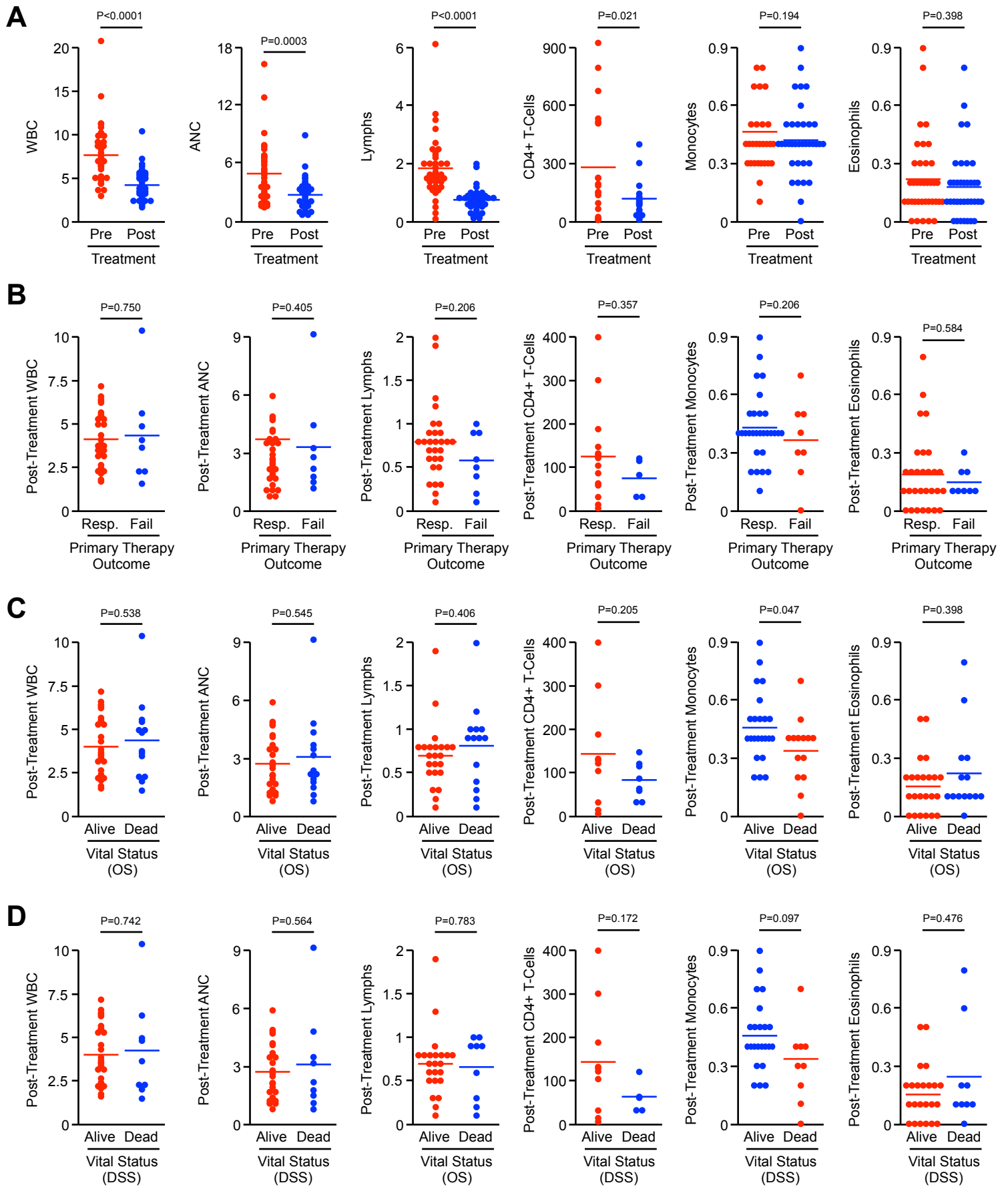

Supplement: Supplementary file 1 — Additional file 1: Figure S1. Demographic information related to clinical outcomesin the anal SCC cohort. Figure S2. Natalsex does not relate to clinical outcomes in the anal SCC cohort. Figure S3. Clinical outcomes arrangedby tumor stage. Figure S4. Peripheralleukocyte counts arranged by demographics and tumor. Figure S5. Overall and disease-specific survival arranged bydemographic information. Figure S6.Pre-treatment anal SCCtumors with an inflamed stroma and/or increased neutrophil infiltrating areassociated with clinical outcomes. Figure S7. Overalland disease-specific survival arranged by demographic information. Supplemental Dataset S1. Pre-treatmentlab values, pre- and post-treatment histology results, and select demographicinformation for each individual patient. TableS1. Individualized clinical characteristics of anal SCC cohort. Table S2. Clinical characteristics ofanal SCC patient cohort arranged by clinical outcome. [file 12885_2022_9742_MOESM1_ESM.zip › 12885_2022_9742_MOESM1_ESM/Figure S7.pdf]
